# MAOA-a novel decision maker of apoptosis and autophagy in hormone refractory neuroendocrine prostate cancer cells

**DOI:** 10.1038/srep46338

**Published:** 2017-04-12

**Authors:** Yi-Cheng Lin, Yi-Ting Chang, Mel Campbell, Tzu-Ping Lin, Chin-Chen Pan, Hsin-Chen Lee, Jean C. Shih, Pei-Ching Chang

**Affiliations:** 1 Institute of Microbiology and Immunology, National Yang-Ming University, Taipei, Taiwan; 2 Institute of Pharmacology, National Yang-Ming University, Taipei, Taiwan; 3 Department of Pharmacology and Pharmaceutical Sciences, School of Pharmacy, University of Southern California, Los Angeles, CA 90089, USA; 4 UC Davis Cancer Center, University of California, Davis, CA 95616, USA; 5 Institute of Clinical Medicine, National Yang Ming University, Taipei, Taiwan; 6 Department of Urology, School of Medicine, and Shu-Tien Urological Research Center, National Yang-Ming University, Taipei, Taiwan; 7 Department of Urology, Taipei Veterans General Hospital, Taipei, Taiwan; 8 Department of Pathology, Taipei Veterans General Hospital, National Yang-Ming University, Taipei, Taiwan; 9 USC-Taiwan Center for Translational Research, University of Southern California, Los Angeles, CA 90089, USA; 10 Department of Cell and Neurobiology, Keck School of Medicine, University of Southern California, 1975 Zonal Ave., Los Angeles, CA 90033, USA; 11 Center for Infectious Disease and Cancer Research, Kaohsiung Medical University, Kaohsiung, Taiwan

## Abstract

Autophagy and apoptosis are two well-controlled mechanisms regulating cell fate. An understanding of decision-making between these two pathways is in its infancy. Monoamine oxidase A (MAOA) is a mitochondrial enzyme that is well-known in psychiatric research. Emerging reports showed that overexpression MAOA is associated with prostate cancer (PCa). Here, we show that MAOA is involved in mediating neuroendocrine differentiation of PCa cells, a feature associated with hormone-refractory PCa (HRPC), a lethal type of disease. Following recent reports showing that NED of PCa requires down-regulation of repressor element-1 silencing transcription factor (REST) and activation of autophagy; we observe that MAOA is a novel direct target gene of REST. Reactive oxygen species (ROS) produced by overexpressed MAOA plays an essential role in inhibiting apoptosis and activating autophagy in NED PCa cells. MAOA inhibitors significantly reduced NED and autophagy activation of PCa cells. Our results here show MAOA as a new decision-maker for activating autophagy and MAOA inhibitors may be useful as a potential therapy for neuroendocrine tumors.

Prostate cancer (PCa) is the second leading cause of cancer death among men in Western countries and its incidence is climbing worldwide^1^. This increase may due to the global aging trends since PCa incidence is closely related to age^2^. Androgen deprivation therapy (ADT) alone or combined with radio- and/or chemotherapy has long been used as treatment of choice for PCa^3^. However, over time a significant population of patients loses responsiveness to ADT and develops hormone-refractory prostate cancer (HRPC)^4^. Due to the improvement of healthcare, patients now live long enough to develop HRPC and in spite of aggressive treatments, the mortality rate of HRPC is always high^5^. One important feature of HRPC is its association with neuroendocrine differentiation (NED)^6^,^7^. Neuroendocrine (NE)-like PCa cells are very difficult to kill and secret cytokines to sustain tumor growth^8^. Therefore, NED has been believed to be a root cause for androgen-independence and high chemoresistance of HRPC. Understanding the NED process may aid the development of intervention strategies designed to delay or prevent the recurrence of HRPC. Recently, NED inducers were identified. These triggers include androgen deprivation^9^,^10^, IL-6 treatment^11^,^12^ and hypoxia^13^,^14^. Interestingly, these reports showed that reduction of repressor element-1 (RE-1) silencing transcription factor (REST) is crucial for NED induction by various inducers^11^,^14^,^15^,^16^ and therefore identified REST as a key repressor for NED of PCa cells.

REST, also known as neuron restrictive silencing factor (NRSF), was originally identified as a critical transcription repressor that silences neuronal gene expression in neural progenitor and non-neuronal cells. Our recent reports show that knockdown of REST may induce NED through activation of autophagy^11^,^14^, a pathway that eukaryotic cells use to degrade long-lived proteins and organelles in response to stress^17^. However, little is known about the underlying mechanisms for REST-mediated autophagy activation. To study this, we performed a genomic ChIP-seq analysis for REST binding and identified a mitochondrial outer membrane protein monoamine oxidase A (MAOA) as a novel REST target gene.

MAOA was originally identified as a mitochondrial outer membrane-bound enzyme that catalyzes oxidative deamination of monoamine neurotransmitters and subsequently generates hydrogen peroxide (H_2_O_2_) as a catalytic byproduct^18^,^19^. It has, therefore, long been believed that MAOA is responsible for maintenance of neurotransmitter homeostasis^18^ and increased oxidative stress (H_2_O_2_) by dysregulation of MAOA is associated with various neurodegenerative diseases^19^,^20^. Emerging evidence has shown the role of MAOA in mediating growth-factor withdraw-21,22,23, mitochondria toxin-24, and neuron toxin-induced^25^ apoptosis of neuronal cells. Though H_2_O_2_ produced by MAOA^26^ has been implicated in most cases of apoptosis, toxin-MAOA interaction mediated opening of mitochondrial permeability transition pore^25^ has been identified as another potential mechanism for cell death. Inhibition of excessive MAOA activity by specific inhibitors protects neuronal death^19^,^20^ and has been widely studied in treating psychiatric and neurological disorders^27^.

Although MAOA was initially identified as a neurotransmitter regulator, recent studies revealed its unanticipated roles in tumorigenesis. However, both tumor suppression and promotion characteristics of MAOA have been identified. Down-regulation of MAOA was associated with cancers including cholangiocarcinoma, esophageal squamous cell carcinoma and hepatocellular carcinoma^28^,^29^. In general, MAOA functions as a tumor suppressor by reducing biogenic amines that stimulate tumor progression through increasing their degradation^28^. In contrast, up-regulation of MAOA was identified in high-grade carcinomas, such as renal cell carcinoma^30^ and PCa^31^,^32^. MAOA plays an oncogenic role by increasing intracellular oxidative stress. Our recent report showed that overexpression of MAOA not only induces epithelial-to-mesenchymal transition (EMT) to enhance invasion and metastasis of PCa cells^33^ but also promotes tumor growth^33^,^34^. Interestingly, inhibition of MAOA by MAOA inhibitors, drugs administered for neurological diseases, not only reduces proliferation^35^,^36^,^37^ and increases apoptosis^36^ of PCa cells but also inhibits xenograft tumor growth^33^,^35^,^38^ and metastasis^33^ in mice.

In this study, we showed that the up-regulation of MAOA occurs in conjunction with REST down-regulation in PCa cells under androgen deprivation conditions. Interestingly, we found that ROS produced by MAOA inhibits apoptosis in a p53-dependent manner and activates autophagy through a p53-independent pathway. Our data show that in addition to general autophagy, MAOA overexpression activates mitophagy, a selective type of autophagy which removes dysfunctional mitochondria. Though ROS and many mitochondria outer membrane proteins have been implicated as causal or contributory factors for mitophagy activation, the underlying mechanisms and target proteins, however, remain largely unknown. This is the first study that investigates the expression and regulation of MAOA in human NE PCa cells. Most importantly, we elucidated that MAOA, a mitochondrial outer membrane-bound enzyme that produces H_2_O_2_, functions as a key regulator in a cell fate-decision by inhibiting apoptosis and activating autophagy. Moreover, a MAOA inhibitor currently used in treating depression showed efficacy in inhibiting androgen deprivation-induced NED in PCa cells. Our results indicate that targeting MAOA may be a potential useful addition to combination therapy in treating different types of NE tumors.

## Results

### MAOA is a novel target of REST and is up-regulated in NE differentiated PCa cells

**Figure 1:**
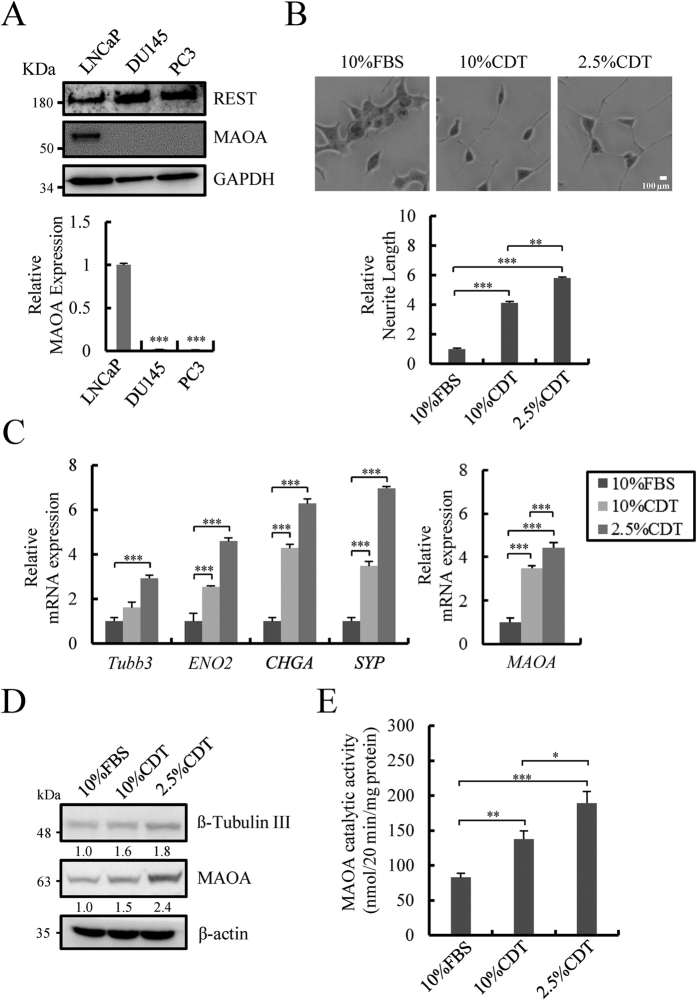
MAOA expression and activity increases in androgen deprivation-induced NE differentiated LNCaP cells. (**A**) LNCaP, PC3, and DU145 were examined for REST and MAOA protein expression by immunoblotting (upper panel). GAPDH was used as control. MAOA mRNA was analyzed by RT-qPCR (lower panel). Data represent mean ± S.D. (n = 3); ***p < 0.001 by *Student’s-t* test. Uncropped images are presented in Supplementary Figure S9A. (**B**) Representative photos (50x magnification) of LNCaP cells cultured with 10% FBS, 10% or 2.5% charcoal/dextran-treated FBS (CDT) for 96 hours (upper panel). Average neurite elongation was quantified from 150 to 250 cells in random fields of three independent experiments (lower panel). **p < 0.01, ***p < 0.001 by *Student’s-t* test. (**C**) The expression of NE markers (left panel) and MAOA (right panel) in LNCaP cells treated as described in (**A**) was analyzed by RT-qPCR and normalized with β-actin. Similar results were obtained using 18 S rRNA as internal control. Data represent mean ± S.D. (n = 3); ***p < 0.001 by *Student’s-t* test. (**D**) Immunoblotting of NE marker β-tubulin III and MAOA in LNCaP cells treated as described in (**B**). β-actin was used as loading control. Uncropped images are presented in Supplementary Figure S9B. (**E**) MAOA activity was measured in LNCaP cells treated as described in (**A**) using an isotopic MAOA catalytic activity assay. Data represent mean ± S.D. (n = 3); *p < 0.05, **p < 0.01, ***p < 0.001 by *Student’s-t* test.

To study the potential role of MAOA in NE PCa cells, three prostate carcinoma cell lines, LNCaP (androgen dependent), PC3 and DU145 (androgen independent) that can be induced for NED were tested^39^. Although limited MAOA expression was observed in PC-3 and DU145 cells, there was a readily detectable level of MAOA in LNCaP cells (Fig. 1A). REST was present in all three cell lines (Fig. 1A). Expression of androgen receptor (AR) and its target gene prostate-specific antigen (PSA) was also analyzed in LNCaP under 10% and 2.5% charcoal/dextran-treated FBS (CDT, androgen deprivation) conditions. A significant reduction of AR and PSA was identified after CDT treatment for 96 hours (Supplementary Fig. S1), however a low level of AR remains under both CDT conditions suggesting low androgen signaling may contribute to NED. Based on these results, androgen dependent LNCaP cells were used in this study. The expression of MAOA under androgen deprivation treatment was also analyzed in LNCaP cells. Consistent with previous reports^9^,^10^, neurite extension was enhanced in LNCaP cells after CDT treatment for 96 hours (Fig. 1B). An increase in mRNA expression of MAOA together with NE markers including, β-tubulin III (TUBB3), neuron specific enolase (ENO2), chromogranin A (CHGA), and synaptophysin (SYP) analyzed by reverse transcription -quantitative PCR (RT-qPCR) was observed 96 hours after both 10% and 2.5% CDT treatment (Fig. 1C). Consistent with the RNA expression analysis, the protein level of MAOA and β-tubulin III was increased under CDT treatment (Fig. 1D). An increase in MAOA activity was confirmed 96 hours under CDT treatment, to a higher extent in the 2.5% CDT condition (Fig. 1E). The temporal co-up-regulation of NED markers and MAOA levels support a potential role of MAOA in mediating androgen deprivation-induced NED.

**Figure 2:**
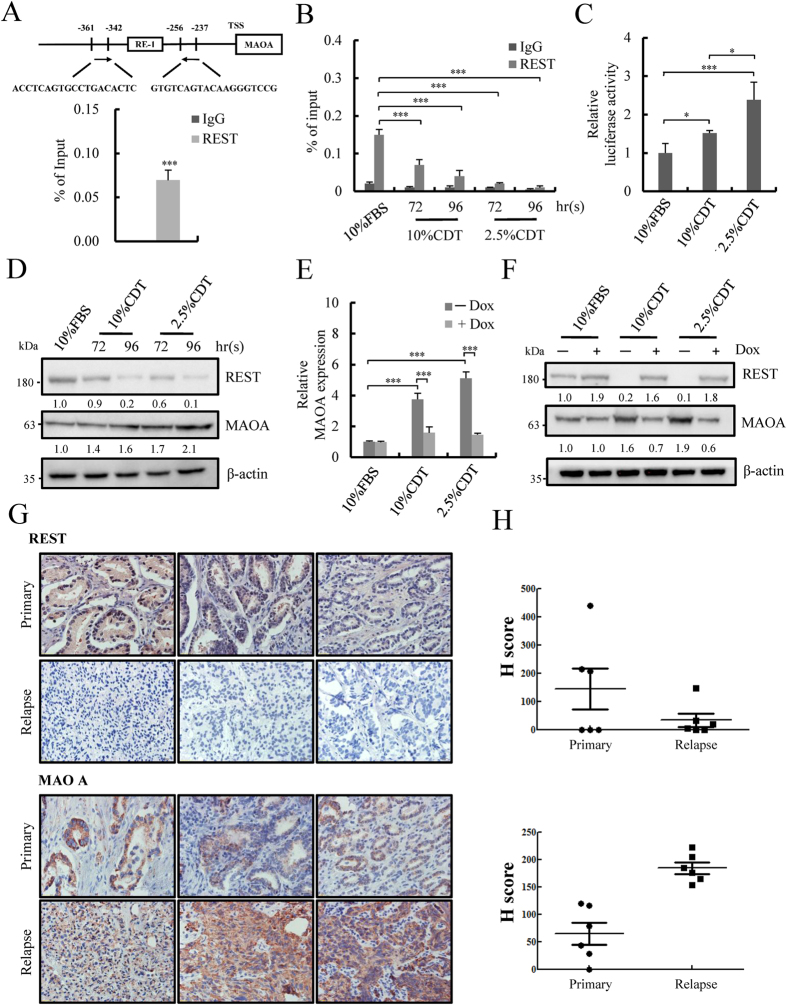
REST directly targets and inhibits MAOA promoter. **(A**) Schematic representation of REST binding site (RE-1 site) identified by ChIP-seq analysis and JASPAR prediction (upper panel) in the promoter region of MAOA. The locations of qPCR primers (short bars) are indicated. ChIP was performed using anti-REST antibody and chromatin prepared from LNCaP cells. REST binding to MAOA promoter region was analyzed by real-time qPCR. Data represent mean ± S.D. (n = 3); ***p < 0.001 by *Student’s-t* test. (**B**) LNCaP cells were cultured in phenol-red free medium supplied with 10% FBS, 10% or 2.5% CDT for 72 and 96 hours. ChIP-qPCR assay shows that REST binding on MAOA promoter, and this binding is reduced by CDT treatment. The enrichment of amplicon was normalized to input. Data represent mean ± S.D. (n = 3); ***p < 0.001 by *Student’s-t* test. (**C**) Luciferase reporter assays were performed by transfecting LNCaP cells with a reporter construct containing the MAOA promoter. At 24 hours post-transfection, cells were incubated with 10% or 2.5% CDT for 96 hours and the luciferase activities were measured. Data represent mean ± S.D. (n = 3); *p < 0.05, ***p < 0.001 by *Student’s-t* test. (**D**) Immunoblotting of REST and MAOA in LNCaP cells treated as described in (**C**). β-actin was used as loading control. Uncropped images are presented in Supplementary Figure S10A. (**E**) The mRNA level of MAOA in LNCaP-TR-REST cells treated as described in (**C**) for 96 hours in the presence ( + ) or absence (−) of Dox was analyzed by RT-qPCR and normalized with β-actin. ***p < 0.001 by *Student’s-t* test. (**F**) Immunoblotting of REST and MAOA in cells treated as in (**F**). β-actin was used as loading control. Uncropped images are presented in Supplementary Figure S10B. (**G** and **H**) Immunohistochemistry (IHC) analysis of REST and MAOA in six primary and six relapsed PCa specimens. Images are representative (magnification, x40) (**G**) and quantification based on their *H*-score. Data are average with S.D. from 6 specimens in each group (**H**). *p < 0.05, **p < 0.01 by *Student’s-t* test.

Using binding site prediction and ChIP-seq assay of REST performed on REST-overexpressing CWR22Rv1 dells, we identified a potential binding site for REST in the MAOA promoter (Fig. 2A, upper panel), a transcriptional repressor that inhibits NED of PCa cells^11^,^14^,^15^,^16^. ChIP-qPCR of REST performed in LNCaP cells using primers specific for the identified REST binding site confirmed the binding of REST on MAOA promoter (Fig. 2A, lower panel). The binding status of REST to the MAOA promoter was further compared in control medium (10% fetal bovine serum; FBS) and in 10% or 2.5% CDT-treated LNCaP cells by ChIP-qPCR. REST binding was observed in control and this binding was significantly reduced under CDT treatment, with a higher reduction observed under 2.5% CDT conditions (Fig. 2B). The derepression of the MAOA promoter under CDT treatment was analyzed by dual luciferase assays using a MAOA 2-kb promoter-luciferase construct. Consistent with ChIP results, the MAOA promoter activity was significantly increased in CDT-treated cells, with a higher extent in the 2.5% CDT condition (Fig. 2C). Western blot analysis also showed reduced expression of REST, concomitant with increased expression of MAOA under CDT treatment (Fig. 2D). To characterize REST-mediated transcriptional repression of MAOA, the REST-inducible LNCaP-TR-REST cells that we generated previously were used^11^. REST overexpression resulted in a significant inhibition of MAOA expression in response to CDT treatment at both the mRNA (Fig. 2E) and protein (Fig. 2F) level. We further determined the expression of REST and MAOA in primary and relapsed PCa specimens. Basal cytokeratin 5 expression was used to confirm a reduction of basal cells in tumor-containing sections (Fig. S2). Consistently, expression of REST and MAOA was significantly reduced and increased, respectively, in relapsed PCa compared with primary PCa specimens (Fig. 2G and H). These data indicate that down-regulation of REST may contribute to MAOA up-regulation in NE differentiated PCa cells.

### MAOA is crucial for androgen deprivation-induced NED

**Figure 3:**
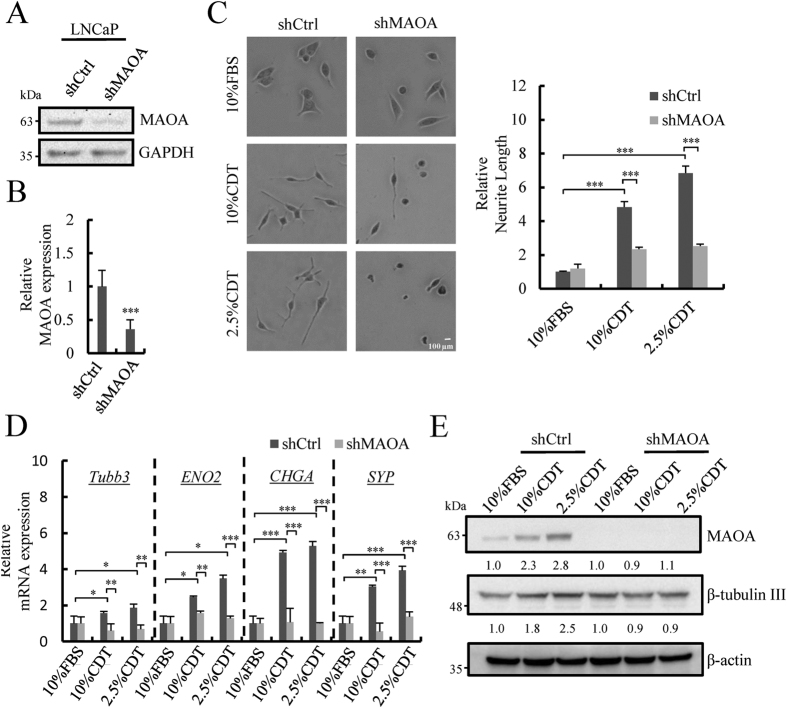
Knockdown of MAOA suppresses androgen deprivation-induced NED. (**A**) Immunoblotting of MAOA in shCtrl and shMAOA LNCaP cells. GAPDH was used as the loading control. Uncropped images are presented in Supplementary Figure S11A. (**B**) The mRNA level of MAOA in control and MAOA knockdown LNCaP cells was assessed by RT-qPCR and normalized with β-actin. ***p < 0.001 by *Student’s-t* test. (**C**) Representative photos (50x magnification) of LNCaP-shCtrl and -shMAOA cells treated with 10% or 2.5% CDT for 96 hours (left panel). Average neurite elongation was quantified as described in Fig. 1B (right panel). ***p < 0.001 by *Student’s-t* test. (**D**) The mRNA levels of NE markers in cells treated as in (**B**) were assessed using RT-qPCR and normalized against β-actin. Similar results were obtained using 18 S rRNA as internal control. Data represent mean ± S.D. (n = 3); *p < 0.05, **p < 0.01, ***p < 0.001 by *Student’s-t* test. (**E**) Immunoblotting analysis of MAOA and β-tubulin III in cells treated as described in (**B**). β-actin was used as loading control. Uncropped images are presented in Supplementary Figure S11B.

To study whether MAOA is involved in androgen deprivation-induced NED, MAOA knockdown LNCaP cells (shMAOA), generated in our previous study was used^33^ (Fig. 3A and B). As shown in left panel of Fig. 3C, MAOA knockdown strongly reduced androgen deprivation-induced neurite extension in LNCaP cells. Quantification of neurite length by MetaMorph showed significant inhibition of this phenotype (Fig. 3C, right panel). The mRNA levels of NE markers in shCtrl and shMAOA cells were determined by RT-qPCR. Knockdown MAOA did not alter the expression of NE markers in control media (Supplementary Fig. S3). However, a significant inhibition of the induction of NE markers in response to MAOA knockdown was observed in cells grown in CDT media (Fig. 3D). Consistently, the increase of β-tubulin III protein expression under androgen deprivation conditions was also abolished in shMAOA cells (Fig. 3E). Together, these data together demonstrate that MAOA may function downstream of REST and is involved in androgen deprivation-mediated NED of PCa cells.

### Up-regulation of MAOA protein protects against androgen deprivation-induced apoptosis in NE differentiated cells

Interestingly, while we were studying the role of MAOA in NED, a morphological change with cell round-up was observed (Supplementary Fig. S4A, left panel). A significant increase in round-up cells was identified in shMAOA cells treated with 10% and 2.5% CDT (Supplementary Fig. S4A, right panel). Consistently, DAPI staining showed an increase in chromatin condensation in shMAOA cells under 10% and 2.5% CDT treatment (Supplementary Fig. S4B). These data suggest a tendency towards increased cell death in NE differentiated PCa cells upon MAOA knockdown.

**Figure 4:**
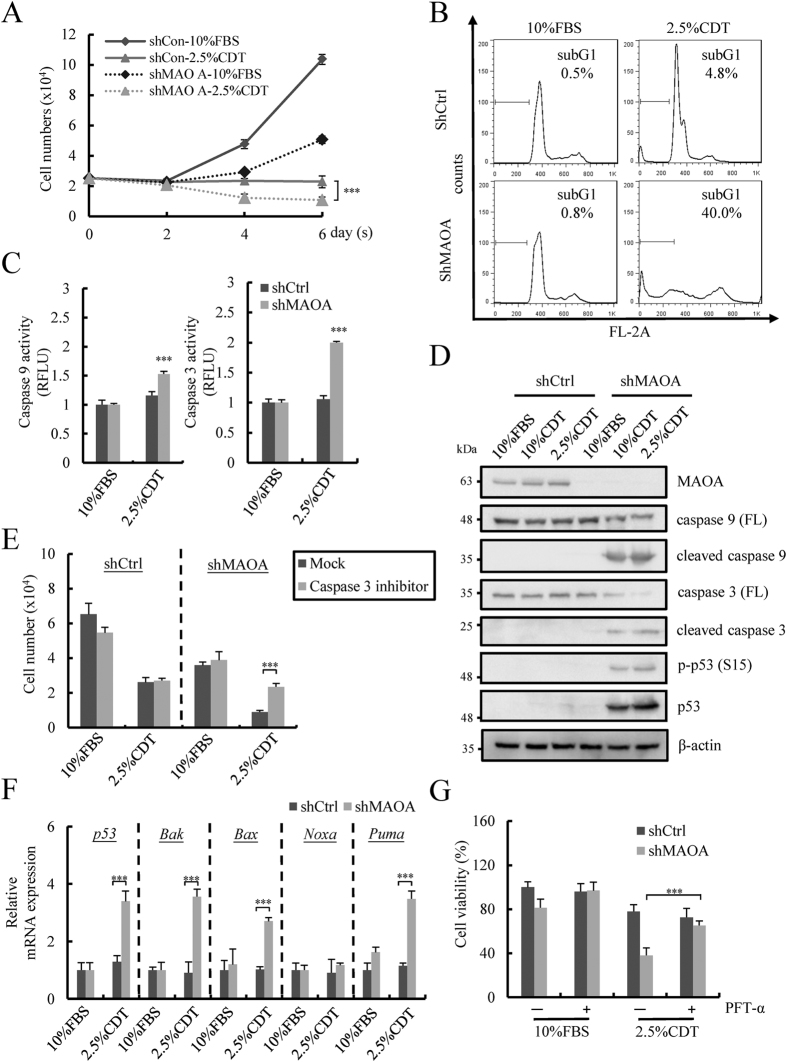
Knockdown of MAOA induces p53-dependent cell apoptosis in NE differentiated LNCaP cells. (**A**) Control and MAOA knockdown LNCaP cells treated with 2.5% CDT was evaluated at the indicated time points using trypan blue dye exclusion assay. Data represent mean ± S.D. (n = 3); ***p < 0.001 by *Student’s-t* test. (**B**) Cells treated as described in (A) for 96 hours were stained with propidium iodide (PI) and measured by flow cytometry. (n = 2) (**C**) Cytosolic caspase 3 and 9 activity was measured using fluorogenic caspase substrates in cells treated as described in (A) for 96 hours. Data represent mean ± S.D. (n = 3); ***p < 0.001 by *Student’s-t* test. (**D**) Immunoblotting of full length (FL) and thec leavage form of caspase 9 and 3, phospho-p53 (Ser15) and p53 in LNCaP-shCtrl and -shMAOA cells treated as described in Fig. 3C. β-actin was used as loading control. Uncropped images are presented in Supplementary Figure S12. (**E**) Inhibition of caspase 3 by 5 μM Q-DEVD-OPh on the growth of cells treated and measured as described in (**C**). Data represent mean ± S.D. (n = 3); ***p < 0.001 by *Student’s-t* test. (**F**) Expression of intrinsic apoptosis pathway genes was examined in cells treated as described in (**A**) using RT-qPCR and normalized with β-actin. Data represent mean ± S.D. (n = 3); ***p < 0.001 by *Student’s-t* test. (**G**) Inhibition of p53 by 20 μM PFT-α increased viability of LNCaP-shMAOA cells treated as in (**C**). Data represent mean ± S.D. (n = 3); ***p < 0.001 by *Student’s-t* test.

Given that MAOA has been implicated with an anti-apoptotic function in PCa cells^34^,^36^ and that MAOA knockdown induced a significant increase in round-up cells (Supplementary Fig. S4A), we hypothesized that overexpression of MAOA may be involved in the anti-apoptotic characteristic of NE-like PCa cells. To study this, we first analyzed the proliferation of shMAOA and shCtrl cells in response to 10% and 2.5% CDT treatment. Knockdown MAOA significantly inhibited cell proliferation under control culture conditions (Fig. 4A and Supplementary Fig. S5A). Consistent with previous studies, androgen deprivation treatment induced growth arrest in shCtrl cells (Supplementary Fig. S5A). Knockdown MAOA induced a significant decrease in the number of surviving cells among 10% and 2.5% CDT-treated LNCaP cells (Fig. 4A and Supplementary Fig. S5A). The increase in cell death was found to be largely due to increase in the subG1 population (Fig. 4B and Supplementary Fig. S5B) and enhanced apoptotic activity, as evidenced by the significant increase in caspase 9 and 3 activity (Fig. 4C and Supplementary Fig. S5C). Decreased full length protein and increased cleavage forms of caspase 9 and 3 (Fig. 4D) was present at 96 hours in CDT plus MAOA knockdown treatment. To confirm that the observed cell death is caused by the activation of caspase 3, the caspase 3 inhibitor Q-DEVD-OPh was used. The successful inhibition of caspase 3 activation was first verified (Supplementary Fig. S5D). Following this, inhibition of caspase 3 activity significantly increased the surviving cell number of shMAOA cells under CDT treatment (Fig. 4E and Supplementary Fig. S5E). This finding shows for the first time that inhibition of MAOA is able to overcome the apoptosis-resistance of NE differentiated PCa cells.

Given that caspase 9 is a marker for intrinsic apoptosis, we hypothesized a possible role of MAOA overexpression in inhibiting apoptosis through up-regulation of anti-apoptotic gene expression. The expression of intrinsic pro-apoptotic proteins Bax, Bak, Noxa, and Puma^40^ was then further analyzed. Knockdown MAOA significantly increased the expression of Bax, Bak and Puma under androgen derivation conditions (Fig. 4F and Supplementary Fig. S5F). Since Bax, Bak and Puma are target genes of p53^40^, expression of p53 was also analyzed. Consistently, expression of p53 was significantly increased in shMAOA cells under androgen deprivation conditions and the phosphorylation on ser15 accompanied p53 induction (Fig. 4D). These data indicate that p53-mediated apoptosis signaling may account for the death of NE differentiated PCa cells after knockdown of MAOA. To further confirm this concept, Pifithrin-α (PFT-α), a p53 inhibitor, was used. First, a dose-response study was undertaken to determine the concentration of PFT-α (Supplementary Fig. S5G). Consistent with our hypothesis, PFT-α rescued the viability of shMAOA cells under androgen derivation condition (Fig. 4G and Supplementary Fig. S5H). Together, our data here show that MAOA overexpression inhibits the p53-dependent apoptosis in a PCa cell line in response to androgen deprivation-induced NED.

### MAOA is essential for autophagy activation in NE differentiated PCa cells

**Figure 5:**
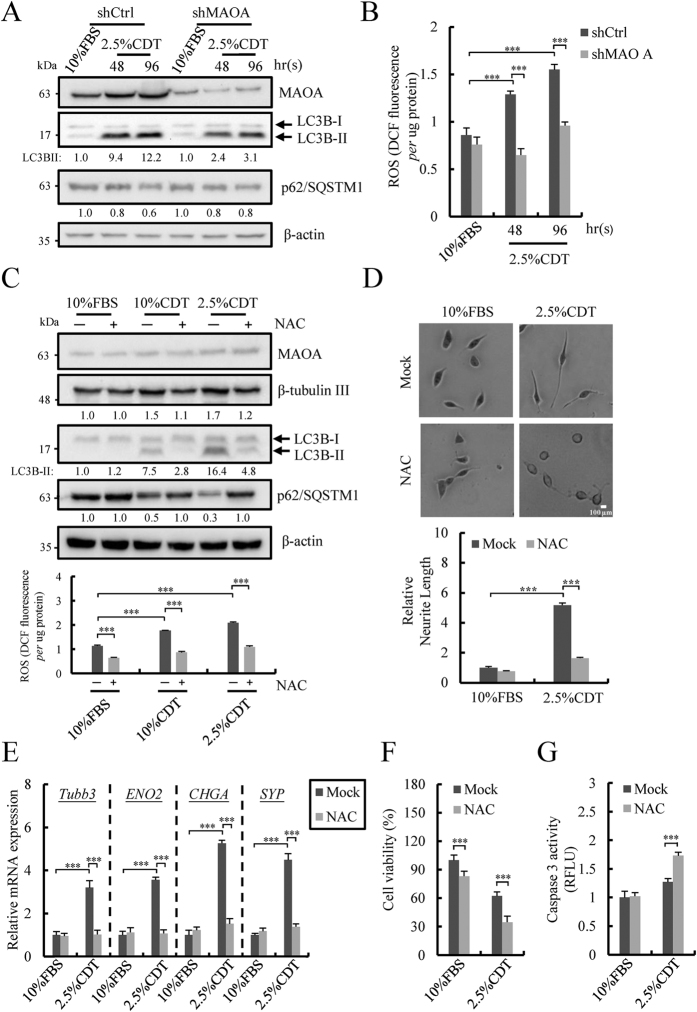
ROS produced by up-regulated MAOA plays an essential role for androgen deprivation-induced autophagy activation, NED and anti-apoptosis. (**A**) Immunoblotting of MAOA, LC3B-II and p62/SQSTM1 in shCtrl and shMAOA LNCaP cells treated as described in Fig. 3C for 48 and 96 hours. β-actin was used as loading control. Uncropped images are presented in Supplementary Figure S13A (right). (**B**) ROS production measured by OxiSelect *In Vitro* ROS/RNS Assay Kit in cells treated as described in (**A**). Data represent mean ± S.D. (n = 3); ***p < 0.001 by *Student’s-t* test. (**C**) Immunoblotting analysis of MAOA, β-tubulin III, LC3B-II and p62/SQSTM1 in cells treated as described in Fig. 1B in the presence or absence of 5 mM NAC. β-actin was used as loading control (upper panel). ROS production was measured as in (**B**). Data represent mean ± S.D. (n = 3); ***p < 0.001 by *Student’s-t* test (bottom panel). Uncropped images are presented in Supplementary Figure S13B. (**D**) Inhibition of ROS reduces neurite extension of LNCaP cells treated as described in (**C**) for 96 hours. Average neurite elongation was quantified as described in Fig. 1B ***p < 0.001 by *Student’s-t* test. (**E**) Inhibition of ROS decreases expression of NE markers in LNCaP cells treated as described in (**C**) for 96 hours. Data represent mean ± S.D. (n = 3); ***p < 0.001 by *Student’s-t* test. (**F** and **G**) Inhibition of ROS decreased cell viability (**F**) and increased caspase 3 activity (**G**) of LNCaP cells treated as described in (**C**). Data represent mean ± S.D. (n = 3); ***p < 0.001 by *Student’s-t* test.

Recent reports including ours showed that autophagy activation is crucial for NED of PCa cells^11^,^12^. The up-regulation of MAOA and its essential role in NED (Fig. 3) prompted us to hypothesize that there may exist a previously uncharacterized relationship between MAOA and autophagy activation. Therefore, autophagy activation under androgen deprivation conditions was analyzed and compared in shCtrl and shMAOA cells. Consistent with previous reports, androgen deprivation-induced autophagy activation was demonstrated by the increase in microtubule-associated protein 1 light chain 3B II (LC3B-II) and decrease in p62/SQSTM1 (Fig. 5A). Consistent with our hypothesis, MAOA knockdown significantly reduced androgen deprivation-induced autophagy activation as shown by a decrease in LC3B-II and increase in p62/SQSTM1compared with control (Fig. 5A and Supplementary Fig. S6A). These data suggest an essential role of MAOA in activation of the autophagy pathway.

### ROS produced by MAOA at the crossroads of apoptosis and autophagy in NE differentiated PCa cells

Increasing evidence has demonstrated that ROS, a group of molecules including H_2_O_2_, are the executioners for stress induced apoptosis^41^ and autophagy^42^. Since MAOA produces H_2_O_2_ as an enzyme by-product and our results here showed that MAOA was involved in both inhibition of apoptosis and induction of autophagy in NE differentiated PCa cells, we hypothesized that ROS produced by MAOA may determine the fate of NE cells towards an anti-apoptotic and autophagy-induction state. To study this, ROS production in shCtrl and shMAOA cells was first analyzed. Indeed, ROS production is increased in control cells under androgen deprivation conditions and this increase was abolished by knockdown MAOA (Fig. 5B and Supplementary Fig. S6B). These data show that the ROS pool produced is contributed via the overexpressed MAOA. To determine whether ROS is essential for androgen deprivation-induced autophagy activation and NED, intracellular ROS was inhibited by N-acetyl cysteine (NAC). Consistent with our hypothesis, NAC treatment for 96 hours reduced both androgen deprivation-induced autophagy activation as evaluated by the decrease in LC3B-II and increase in p62/SQSTM1, and NED as shown in the reduction of β-tubulin III (Fig. 5C). The inhibition of NED by NAC was further confirmed by measurements of neurite extension (Fig. 5D and Supplementary Fig. S6C) and the expression of NE markers (Fig. 5E and Supplementary Fig. S6D). Following the observation of autophagy activation, the role of ROS in inhibiting apoptosis was also analyzed. In agreement with our prediction, ROS inhibition significantly reduced cell viability (Fig. 5F and Supplementary Fig. S6E) by increasing apoptotic activity (Fig. 5G and Supplementary Fig. S6F). Our data here showed that ROS produced by MAOA mediates autophagy activation and apoptosis inhibition in androgen deprivation-induced NE differentiated PCa cells. These results indicate that MAOA may be one of the intrinsic pathway regulators for cell fate decisions.

### MAOA up-regulation under androgen deprivation treatment induces mitophagy

**Figure 6:**
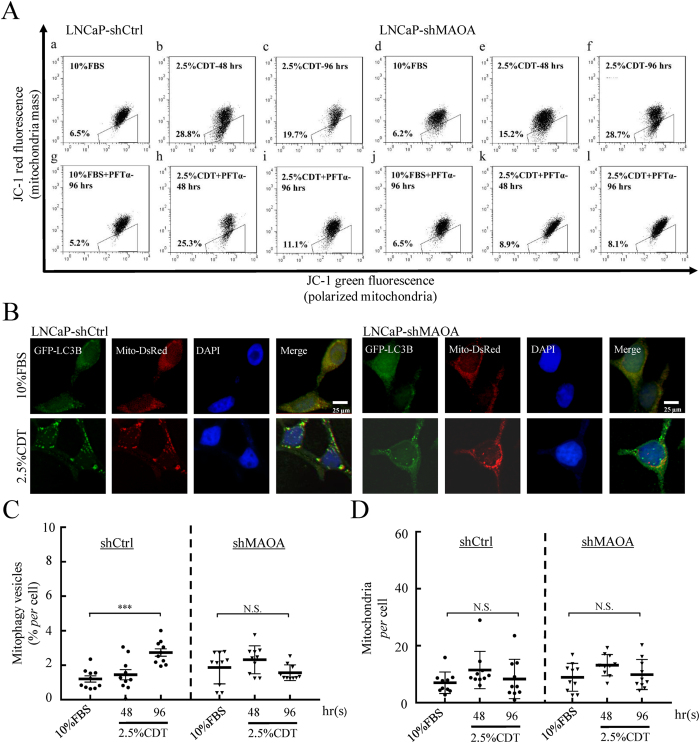
Effect of MAOA on the loss of mitochondrial membrane potential and mitophagy activation of androgen deprivation-induced NE differentiated LNCaP cells. (**A**) Flow cytometry plots of the JC-1 staining. LNCaP-shCtrl and shMAOA cells were treated as in Fig. 3C and stained with JC-1. JC-1 fluorescence was measured in FL-1 (green, representing the total mitochondrial mass) and FL-2 (red, representing polarized mitochondria) channels. JC-1 fluorescence was detected in both FL-1 and FL-2 channels in the majority of shCtrl and shMAOA cells cultured in 10% FBS, with a small percentage of cells showing reduced red fluorescence (a and d). 2.5% CDT treatment increased the number of cells devoid of red fluorescence (b, c, e and f), indicating loss of mitochondrial membrane potential. PFT-α (20 μM) does not affect the number of cells that loss of red fluorescence in 10% FBS (g and j). PFT-α reduced the number of shMAOA cells devoid of red fluorescence (k and l) but not, or to a much less extent, in shCtrl cells (h and i). (n = 3) (**B**) Representative fluorescence images of LNCaP-shCtrl and shMAOA cells stably expressing GFP-LC3B, transiently transfected with mito-DsRed plasmid and treated by 2.5% CDT for 96 hours. Mitophagy was determined by assessing colocalization of GFP puncta, representing autophagosomes, and RFP puncta, representing mitochondria, using fluorescence microscopy. GFP and RFP double positive puncta with 0.8 to 1.3 μm were counted as mitophagy-positive cells. (**C** and **D**) Quantification of the percentage of GFP (autophagosome) and RFP (mitochondrial) colocalized puncta in RFP puncta (**C**) and mitochondrial counts per cell (**D**) in cells treated as in (**B**). Data represent mean ± S.E.M. Average value was quantified from 170 cells in random fields of three independent experiments. N.S., not significant, ***p < 0.001 by *Student’s-t* test.

In addition to regulation of autophagy, increasing evidence has shown that ROS-induced mitochondrial depolarization initiates mitophagy, a selective type of autophagy in which dysfunctional mitochondria are removed by targeting to autophagic degradation^43^. We therefore hypothesized that in NE differentiated PCa cells, MAOA overexpression may induce mitophagy through ROS production. Mitochondrial membrane potential was measured by JC-1 staining. The uncoupling agent carbonyl cyanide 3-chlorophenyl hydrazone (CCCP) was used as positive control (Supplementary Fig. S7A, upper panel). Interestingly, LNCaP cells treated with CDT showed significant increases in mitochondrial depolarization at 48 hours which was reduced at 96 hours post-treatment (Fig. 6A; b,c and Supplementary Fig. S7A; b,c). On the other hand, MAOA knockdown resulted in a delayed but continued increased in depolarized mitochondria in CDT-treated LNCaP cells (Fig. 6A; e, f and Supplementary Fig. S7A; e,f). By using PFTα, we showed that mitochondrial depolarization induced in control cells is p53-independent (Fig. 6A; h,i). However, the depolarized mitochondria in shMAOA cells is p53-dependent as the mitochondrial depolarization was completely abolished by adding PFTα (Fig. 6A; k,l). Since MAOA knockdown can induce p53 expression which consequently promotes cell apoptosis (Fig. 4), the data indicate a direct role for p53 in activating apoptosis in shMAOA cells (Fig. 6A; k,l). In contrast, when MAOA is expressed, mitophagy may be activated and depolarized mitochondria may be removed (Fig. 6A; h,i).

To further confirm our hypothesis, mitophagy was assessed by analyzing colocalization of autophagosomes and mitochondria markers in shCtrl and shMAOA cells. Consistently, significantly more colocalization of autophagosomes and mitochondria was observed in control cells treated with 10% and 2.5% CDT for 96 hours (Fig. 6B,C and Supplementary Fig. S7B, C). Quantification data showed no significant difference in mitochondria number in shCtrl and shMAOA cells (Fig. 6D and Supplementary Fig. S7D). This data support the potential of induction of mitophagy in MAOA expressing NE differentiated PCa cells.

### Application of MAOA inhibitors, pargyline and phenelzine, inhibit androgen deprivation-induced NED

**Figure 7:**
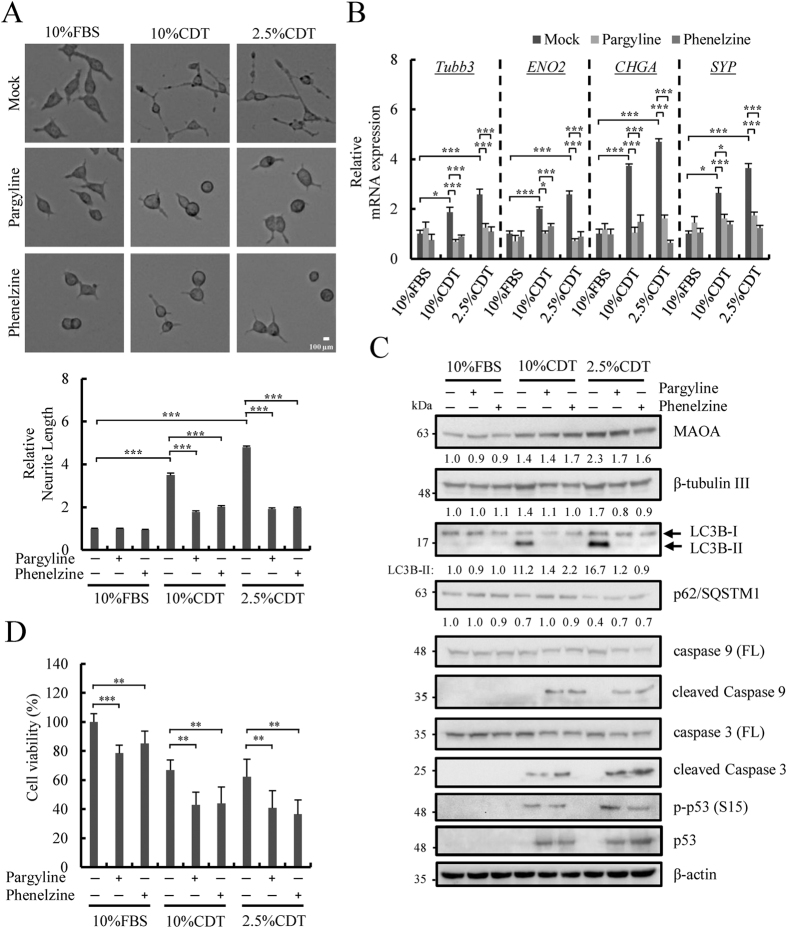
MAOA inhibitors inhibit androgen deprivation-induced NED. (**A**) Pargyline (1 μM) and phenelzine (1 μM) were added in LNCaP cells treated as described in Fig. 1B. Representative photos (50x magnification) are shown (top panel) and the average neurite elongation was quantified as in Fig. 1B (bottom panel). ***p < 0.001 by *Student’s-t* test. (**B**) Expression of NE markers was examined in cells treated as described in (**A**) using RT-qPCR and normalized with β-actin. Data represent mean ± S.D. (n = 3); *p < 0.05, ***p < 0.001 by *Student’s-t* test. (**C**) Immunoblotting of MAOA, β-tubulin III, LC3B-II, p62/SQSTM1, full length (FL) and the cleavage form of caspase 9 and 3, phospho-p53 (Ser15) and p53 in LNCaP cells treated as in (**A**). β-actin was used as loading control. Uncropped images are presented in Supplementary Figure S14. (**D**) Inhibition of MAOA by pargyline (1 μM) or phenelzine (1 μM) decreased viability of LNCaP cells treated as in (**A**). Data represent mean ± S.D. (n = 3); **p < 0.01, ***p < 0.001 by *Student’s-t* test.

We further treated LNCaP cells maintained in control or CDT medium with pargyline^44^ or phenelzine^39^, two potent small-molecule inhibitors of MAOA. The successful inhibition of MAOA activity by pargyline and phenelzine was first confirmed (Supplementary Fig. S8). Consistent with the knockdown experiment, treatment of LNCaP cells with MAOA inhibitors suppressed androgen deprivation-induced neurite extension (Fig. 7A) and expression of NE markers (Fig. 7B,C). Consistently, inhibiting autophagy by MAOA inhibitors also resulted in a decrease in LC3B-II and increase in p62/SQSTM1 (Fig. 7C). Moreover, induction of apoptosis by MAOA inhibitors was observed by a decrease in full length protein and increased in cleaved form of caspase 9 and 3 (Fig. 7C). Consistently, reduced cell viability in cells under CDT treatment by MAOA inhibitors was also observed (Fig. 7D). Taken together, these data demonstrate that MAOA is a key determinant for PCa NED and the potential use of MAOA inhibitors in preventing NED of PCa cells.

## Discussion

The crosstalk between “self-eating” autophagy, and “self-killing” apoptosis, is complex. In most cases, autophagy acts as a cytoprotective mechanism that constitutes a stress adaptation and suppresses apoptosis. However, in specific cellular settings, autophagy establishes an alternative apoptosis signaling, type II programed cell death^45^. Autophagy and apoptosis share common upstream signals and regulators, and exhibit either mutual cooperation or mutual inhibition^45^. This suggests that autophagic and apoptotic effectors are differentially regulated. However, current knowledge on the cellular decision making process between autophagy and apoptosis is still in its infancy.

Common upstream stress mediators of autophagy and apoptosis include ROS, ceramide, and p53 activation. Components of the intracellular milieu contribute to “deciding” between autophagy and apoptosis. For example, sphingosine-1 phosphate inhibits apoptosis induced by ceramide, a well-established inducer for both apoptosis and autophagy, but induces autophagy^46^. Similar to ceramide, ROS induce apoptosis by reducing the mitochondrial membrane potential as well as stimulate autophagy by pathways such as activation of AMPK signaling, disruption of beclin1-bcl-2 interaction, oxidation and inhibition of Atg4, and elimination of damaged mitochondria by mitophagy^47^. However, molecule(s) that contribute to decision-making for ROS are largely unknown.

**Figure 8:**
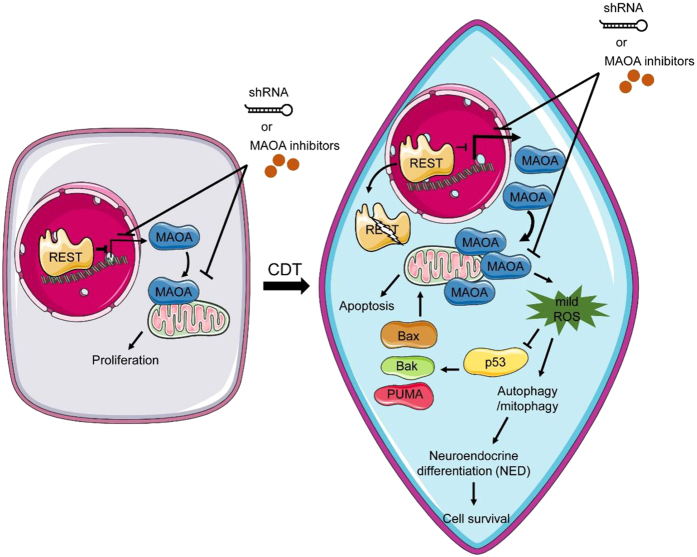
A schematic of our proposed model describing decision making at the crossroads of autophagy and apoptosis in NE differentiated PCa cells. First, MAOA was identified as a novel target gene repressed by REST. Second, ROS (H_2_O_2_) produced by MAOA inhibits p53-mediated apoptosis. Finally and most importantly, ROS produced by MAOA, through a currently unidentified mechanism, activates autophagy, most likely a specific subtype form of autophagy named mitophagy, and consequently induces the autophagy-dependent NED of PCa cells.

It has been long recognized that mitochondrial electron transport chain (mETC) is the main site of production of intracellular ROS. High levels of ROS cause progressive oxidative damage and ultimately cell death. Beside the mETC, low levels of ROS are generated by enzymes such as membrane-bound NADPH oxidase^48^. Different from high-level ROS, low levels of ROS tend to participate in cellular signaling. Consistently, it has been reported that, rather than activate apoptosis, ROS produced by NADPH oxidase inhibit apoptosis^49^. However, the underlying mechanisms have not yet determined. In this study, we found that MAOA, a mitochondrial outer membrane monoamine oxidase that produced H_2_O_2_, functions as a novel “decision maker” between autophagy and apoptosis. ROS produced by MAOA inhibits apoptosis by reducing p53 transactivation function and concomitantly activates autophagy through induction of mitophagy (Fig. 8).

Tumor suppressor p53 is a well-known apoptosis inducer. In general, in response to high levels of ROS signal, p53 protein is phosphorylated, stabilized and activated to induce the expression of BH3-only protein PUMA and NOXA that has been linked to promote mitochondrial outer membrane permeabilization (MOMP) via the BAX and BAK channel^50^. The activation of intrinsic apoptotic pathway is characterized by MOMP and release of cytochrome c from mitochondria that consequently results in assembly of caspase-activation complexes and activation of caspase 9^51^. In addition to phosphorylation, acetylation was recently identified as another post-translational modification (PTM) that activates p53^52^. Emerging evidence has shown that oxidative stress promotes acetylation of p53 that consequently leads to activation of p21 and induction of senescence and apoptosis^53^. However, here in this study, we showed for the first time that ROS produced by MAOA (Fig. 5B and Supplementary Fig. S6B) inhibits apoptosis (Fig. 5F,G and Supplementary Fig. S6E,F). Knockdown MAOA significantly increased p53 and its target genes BAX, BAK and PUMA expression (Fig. 4D,F and Supplementary Fig. S5F). Moreover, the p53 small molecule inhibitor PFTα that suppressed p53 transcription and mitochondrial translocation^54^ blocked MAOA knockdown induced apoptosis (Fig. 4G and Supplementary Fig. S5H). Although we do not fully understand our results, we propose a hypothesis to explain this observation. During oxidative deamination, MAOA increases H_2_O_2_ levels inside the mitochondria that consequently oxidize glutathione to glutathione disulfide (GSSG)^55^ and the accumulated GSSG reacts with thiol groups of p53 and form S-glutathionylated p53^56^. Glutathionylation of p53 by H_2_O_2_ produced by MAOA may result in p53 inactivation. This hypothesis is supported by recent observations including accumulation of p53 in the mitochondrial matrix under oxidative stress^57^ and a report from Velu *et al*. which showed that glutathionylation of p53 during oxidative stress inhibits p53 function^56^. Moreover, inhibition of p53 by glutathionylation may be due to a decrease in the phosphorylated forms of p53 as demonstrated in a recent report of STAT3 which showed that mild oxidative stress induced glutathionylation and reduced phosphorylation of STAT3^58^. However, further studies are necessary to elucidate this hypothesis. Moreover, the difference between cytosolic and mitochondrial H_2_O_2_ in modulating p53 PTM and activation is a very interesting topic worth further study.

Emerging evidence implicates ROS as important signals involved in activation of general autophagy. Interestingly, recent work using CCCP, a mitochondrial-uncoupling reagent, to induce depolarization of entire mitochondrial membrane was shown to induce mitophagy, a selective form of autophagy that degrades mitochondria. Consistently, using mitochondrial KillerRed (mtKR), a mitochondrial-targeted photosensitizer, to induce short bursts of ROS produced in mitochondrial matrix resulted in loss of mitochondrial membrane potential and subsequently activation of mitophagy^43^. Here, our study showed that ROS produced by MAOA activates mitophagy (Fig. 6B,C and Supplementary Fig. S7B, S7C) and may consequently remove the dysfunctional mitochondria in NE differentiated PCa cells (Fig. 6A and Supplementary Fig. S7A). However, detailed mechanisms that may responsible for MAOA mediated mitophagy await identification. Potential mechanisms include the involvement of molecular mechanisms of PINK/Parkin, NIX/Bnip3L, and FUNDC1 pathways that have been well characterized in mitophagy activation^59^. One common element in all these pathways is its initiation by stabilizing the mitochondrial outer membrane protein component in the mitochondrial outer membrane. Stabilizing any of these proteins in mitochondria by H_2_O_2_ produced from MAOA or through protein-protein interactions with MAOA (a mitochondrial localized protein) is a potential mechanism worth further investigation. In addition, one recent report from Morell *et al*. showed that the expression of LAMP2, a lysosomal membrane protein involved in stabilizing lysosome, was essential for autophagy activity during NED of PCa LNCaP cells^60^. The crosstalk between MAOA and LAMP2 in autolysosomal formation is another potential mechanism worth further investigation. Moreover, as mentioned above, p53 is a key regulator of both autophagy and apoptosis pathways^61^ and its function is highly regulated by PTMs^62^. The potential of MAOA in modulating PTMs of p53 which consequently inhibit apoptosis and activate autophagy is another potential mechanism for future research.

In addition to identifying MAOA as a decision maker at the crossroads of autophagy and apoptosis, another important finding in this study was the identification of MAOA as a novel target gene of REST. REST is a zinc-finger transcription repressor that regulates neuron-specific genes by direct binding to a 21 bp consensus sequence named RE-1 and recruits distinct corepressive complexes. Down-regulation of REST during neurogenesis has long been known. Recent studies including ours revealed unanticipated roles of REST in modulating androgen deprivation-15, hypoxia-13,14 and IL-6-induced-11,63 NED of PCa and which showed that REST expression is significantly reduced in relapsed PCa tissue^11^,^15^. Most interestingly, our previous report showed that REST knockdown alone enhances autophagy activation and that autophagy is involved in NED of PCa cells^11^. Recent reports including ours showed that PCa cells exposed to semi-starvation culture conditions by using 2.5% FBS will also increase autophagy activation^11^,^64^. This may explain the higher effect observed in 2.5% CDT treatment when compared with 10% CDT. Though the essential role of autophagy in NED has been elucidated^11^,^12^ the underlying mechanisms for REST-mediated autophagy activation are still largely unknown. Our study here revealed that MAOA, a mitochondria enzyme that is highly associated with advanced PCa^33^, is directly targeted by REST in PCa cells and this target repression was lost in NE differentiated PCa cells (Fig. 2B). As mentioned above, MAOA overexpression activates autophagy and inhibits apoptosis. Together, these data suggest the involvement of MAOA in REST-mediated autophagy activation and NED in PCa. Since our data presented here demonstrates the up-regulation of MAOA in androgen deprivation-induced NE differentiated PCa cells and one recent study showed the induction of MAOA expression by chemotherapy^34^, we suggest that the addition of MAOA inhibitors may be a potential therapeutic for advanced PCa patients receiving combination ADT and chemotherapy.

It has been long known that mitochondria are the central regulator of the apoptotic intrinsic pathway. Here, we showed that mitochondria are also involved in modulating autophagy activation. A mitochondrial outer membrane protein MAOA was found as a novel gene target of REST, a recently identified regulator for autophagy activation in NE differentiated PCa cells. MAOA was not only involved in modulating autophagy activation but also inhibiting apoptosis in androgen deprivation-induced NE differentiated PCa cells. This suggests MAOA may function as a novel mediator of REST-induced autophagy activation and NED in PCa cells. Our previous report showed that MAOA associates with PCa malignancy and metastasis^33^. Our data here suggest that overexpression of MAOA may also be required for the progression to HRPC. Altogether, MAOA inhibitors that have been used clinically for treating psychological disease may have potential in combination therapy for all types of advanced PCa including castration- and chemo-resistant HRPC.

## Methods

### Cells and reagents

LNCaP cells were maintained in RPMI 1640 supplemented with 10% fetal bovine serum (FBS) at 37 °C under a humidified atmosphere saturated with 5% CO_2_. LNCaP-shCtrl, LNCaP-shMAOA^33^ and LNCaP-TR-REST^11^ cells were generated and maintained as pervious described. For induction of NED, cells were cultured in phenol-red free RPMI 1640 supplemented with 10% or 2.5% charcoal/dextran-treated FBS (Hyclone). p53 inhibitor Pifithrin-α (PFT-α), ROS inhibitor N-acetyl-L-cysteine (NAC), MAO inhibitors of pargyline and phenelzine were purchased from Sigma-Aldrich. Caspase 3 inhibitor Q-DEVD-OPh was purchased from Biovision.

### Chromatin immunoprecipitation-sequencing (ChIP-Seq)

ChIP assays were performed by using the protocol described from the Farnham laboratory (provided at http://genomics.ucdavis.edu/farnham). ChIP grade anti-REST rabbit antibodies (Millipore) and rabbit non-immune serum IgG (Alpha Diagnostic International) were used for IP. Chromatin DNA for ChIP assays was prepared from 4 × 10^8^ CWR22Rv1-TR-REST cells treated with Dox for 72 hours. Sequencing libraries were prepared from 50 ng of ChIPed DNA suspended in 30 μl of ddH_2_O following the protocol from Illumina. 400 bp DNA fragments were subjected to paired-end high throughput sequencing on Illumina^®^ HiSeq 2000. ChIP-Seq data was aligned onto human genome hg19. Approximately 32,046,223 reads were mapped after filtering and quality control (QC). The peak detection method of Partek software (Partek Genomics suite 6.6) was used to identify potential REST binding sites.

### MAOA catalytic activity assay

Total cell lysates (TCLs) were prepared from cells using NaPi buffer [50 mM Sodium-phosphate buffer pH7.4] followed by sonication. Protein concentration was then measured using Bio-Rad protein assay dye reagent following the manufacturer’s protocol (Bio-Rad). 100 μg of TCLs was incubated with 1 mM [^14^C]5-HT in 50 mM sodium phosphate buffer (pH 7.4) at 37 °C for 20 minutes. The reaction was terminated by the addition of 100 μl of ice-cold 6 N HCl. The reaction products were extracted with benzene/ethyl acetate (1:1) and centrifuged (1,100 rpm) at 4 °C for 7 minutes. The organic phase containing the reaction products was extracted and the radioactivity was determined by liquid scintillation spectroscopy.

### Flow Cytometry Analysis

LNCaP-shCtrl and -shMAOA cells were seeded in 6 cm dish at a density of 3 × 10^5^ cells/well and treated with 10% or 2.5% CDT the next day. After 96 hours, the cells were harvested, fixed in 1 ml of ice cold 80% ethanol and stored in −20 °C. Before analysis, the pellet was washed twice with 1 ml of PBS, permeabilized with Triton X-100 PBS buffer [0.1% Triton X-100, 1X PBS, 1 ug/ml RNAse A (Thermo Fisher Scientific)] and stained with 1 mg/ml propidium iodide (PI) (Thermo Fisher Scientific) in dark at room temperature (RT) for 15 minutes. Cells were analyzed using BD^™^ LSR II flow cytometer.

### Assessment of mitochondrial membrane potential

LNCaP-shCtrl and -shMAOA cells treated with 10% or 2.5% CDT for 48 or 96 hours in the presence or absence of PFT-α. Cells were rinsed with PBS and stained with 1 ml RPMI 1640 medium containing 2 μM JC-1 dye (Molecular Probes) for 30 minutes at 37 °C. After staining, cells were washed with ice-cold PBS, resuspended in 1 ml ice-cooled PBS, and assessed by flow cytometry (LSRII, BD). Frequency plots were prepared for FL1 (green fluorescence) and FL2 (red fluorescence) to determine the percentage of the mitochondria stained green (low membrane potential) and red (normal membrane potential).

### Immunohistochemistry staining (IHC staining)

Paraffin-embedded specimens from patients with primary PCa and CRPC that was collected at Taipei Veterans General Hospital (TVGH) were included in this study. Ethics was approved by the TVGC Institute Review Board. Written informed consent was obtained. Tissue sections were deparaffinized in xylene, rehydrated through graded ethanol, antigen was retrieved by microwaving sections in 10 mM citrate buffer (pH 6.0) for 10 minutes, and endogenous peroxidase activity was inactivated with 3% H_2_O_2_ for 10 minutes at RT. Slides were blocked with antibody diluent containing background-reducing components (DAKO, S302281) and then stained overnight at 4 °C with anti-REST (1:200, BETHYL). anti-MAOA (1:200, Abcam) and cytokeratin 5 (1:200, Genetex) antibodies and visualized following standard protocol by using 3-amino-9-ethylcarbazole (AEC) as chromogen (DAKO, K346430) in the presence of hematoxylin counterstaining. Scoring of REST and MAOA expression in human PCa samples was performed by a pathologist (Dr. Pan) and was based on *H*-score, which is derived by multiplying the staining intensity (0–4) with the percentage of epithelial cells with positive staining.

### Immunofluorescence

After transfection of pDs-Red2 mito plasmid (red) into LNCaP-shCtrl and -shMAOA cells expressing GFP-LC3B, cells were re-seeded on poly-L-lysine-coated coverslips (Marienfeld) and then subjected to with 10% or 2.5% CDT treatment. 48 and 96 hours after treatment, cells were fixed with 4% paraformaldehyde, stained with DAPI (Invitrogen), and then mounted in mounting solution (20 mM n-propylgallate, 20% PBS, and 80% glycerol). The images were visualized by a fluorescence microscope (DMI4000B, Leica) with 63x lens. The GFP and RFP punctates (1–4 μm) were analyzed by using Transfluor^®^ in MetaMorph (Molecular Devices).

### Immunoblotting analysis

Total cell lyates (TCLs) were prepared from cells using Triton X-100 lysis buffer [0.5% Triton X-100, 1X PBS, 1X protease inhibitors (Roche, 04693132001)]. 40 μg of TCLs was loaded onto 8–15% SDS-PAGE, transferred to the Hybond-C Extra membranes (Amersham Biosciences), and incubated with antibodies against proteins of interest, including β-tubulin III (1:5000, Cell Signaling Technology), LC3B (1:1000, Cell Signaling Technology), p62/SQSTM1 (1:1000, Cell Signaling Technology), full-length caspase 9 (1:1000, Cell Signaling Technology), cleaved caspase 9 (1:1000, Cell signaling technology), full-length caspase 3 (1:1000, Cell Signaling Technology), cleaved caspase 3 (1:1000, Cell signaling technology), phospho-p53 (Ser15) (1:1000, Cell Signaling Technology), p53 (1:1000, Cell Signaling Technology), REST (1:500, BD), MAOA (1:2000, Santa Cruz) and GAPDH (1:3000, Santa Cruz). The signals were visualized using a Pierce ECL Western Blotting substrate (Thermo Fisher Scientific) and imaged via a ChemiDoc Touch imaging system (Bio-Rad).

### Real time reverse transcription (RT) quantitative PCR (qPCR) analysis

Total RNAs were isolated using TRIzol (Invitrogen) following standard protocol and reverse-transcribed into cDNA by First Strand cDNA Synthesis Kit (Thermo Fisher Scientific). Real-time qPCR was performed using SYBR Green master mix (Bio-Rad) on a Bio-Rad iCycler. Relative gene expression was calculated by the 2 –ΔΔCT formula. Primers are shown in Supplementary Table 1.

### Cell viability assay

5 × 10^3^ cells were seeded on 96-well plates and treated with 10% or 2.5% CDT in the presence or absence of PFT-α, NAC, or MAO inhibitors (pargyline and phenelzine). Cell viability was measured by adding MTS reagent (Promega) for 3 hours at 37 °C. The absorbance was measured spectrophotometrically at 490 nm with a microplate reader (Synergy HTX, Bio-Tek). The culture medium was used as blank control.

### Reactive oxygen species assay

1 × 10^4^ cells were seeded in 96-well clear bottom black plates and treated with 10% or 2.5% CDT to induce NED for 48 or 96 hours. Following the treatments, intracellular ROS levels were determined by using OxiSelect *In Vitro* ROS/RNS Assay Kit (Cell Biolabs) according to the manufacturer’s guideline (Cell Biolabs). Cells treated with H_2_O_2_ were used as a positive control.

### Caspase activity assay

The assay was performed as described in previous study^65^. Briefly, cells were washed once with phosphate buffered saline (PBS) and lysed in reporter lysis buffer (Promega). Different amounts of caspase proteins (100 μg for caspase 3 and 200 μg for caspase 9) were diluted in 250 μl reporter lysis buffer and incubated with equal volumes of 2x caspase reaction buffer containing 100 μM fluorogenic substrates for caspase 3 or 9 (Calbiochem) at 37 °C for 3 hours. The intensity of emitted fluorescence at 505 nm was measured using a fluorescence spectrometer (Bio-Tek).

### Luciferase assay

Cells were cotransfected with MAOA 2-kb promoter luciferase construct^66^ or vector control and TK-Renilla plasmid, maintained in CDT for another 96 hours and harvested to determine the luciferase activities by using a dual-luciferase reporter assay system (Promega). The activity of the firefly luciferase was normalized to that of the Renilla luciferase in the same assayed sample.

### Statistical Analysis

Statistical analysis was performed using *Student’s-t* tests and indicated as N.S., not significant; *p < 0.05; **p < 0.01; ***p < 0.001.

## Additional Information

**How to cite this article:** Lin, Y.-C. *et al*. MAOA-a novel decision maker of apoptosis and autophagy in hormone refractory neuroendocrine prostate cancer cells. *Sci. Rep.* **7**, 46338; doi: 10.1038/srep46338 (2017).

**Publisher's note:** Springer Nature remains neutral with regard to jurisdictional claims in published maps and institutional affiliations.

## Supplementary Material

Supplementary Information
