# Supplementary material for: MAOA-a novel decision maker of apoptosis and autophagy in hormone refractory neuroendocrine prostate cancer cells
Source: Sci Rep. 2017 Apr 12;7:46338. doi: 10.1038/srep46338 (PMC5389346; doi:10.1038/srep46338)
Supplement: Supplementary Information [file srep46338-s1.pdf]

Supplementary information

MAOA-a novel decision maker of apoptosis and autophagy in hormone refractory neuroendocrine prostate cancer cells

Yi-Cheng Lin[1,7,8], Yi-Ting Chang[1], Mel Campbell[2], Tzu-Ping Lin[3, 4, 5], Chin-Chen Pan[6], Hsin-Chen Lee[7], Jean C. Shih[8,9,10§], Pei-Ching Chang[1,11§]

[1]Institute of Microbiology and Immunology, National Yang-Ming University, Taipei, Taiwan
[2]UC Davis Cancer Center, University of California, Davis, CA 95616, USA
[3]Institute of Clinical Medicine, National Yang Ming University, Taipei, Taiwan
[4]Department of Urology, School of Medicine, and Shu-Tien Urological Research Center, National Yang-Ming University, Taipei, Taiwan
[5]Department of Urology, Taipei Veterans General Hospital, Taipei, Taiwan
[6]Department of Pathology, Taipei Veterans General Hospital, National Yang-Ming University, Taipei, Taiwan
[7]Institute of Pharmacology, National Yang-Ming University, Taipei, Taiwan
[8]Department of Pharmacology and Pharmaceutical Sciences, School of Pharmacy, University of Southern California, Los Angeles, CA 90089, USA.
[9]USC-Taiwan Center for Translational Research, University of Southern California, Los Angeles, CA 90089, USA.
[10]Department of Cell and Neurobiology, Keck School of Medicine, University of Southern California, 1975 Zonal Ave., Los Angeles, CA 90033, U.S.A.
[11]Center for Infectious Disease and Cancer Research, Kaohsiung Medical University, Kaohsiung, Taiwan

*Corresponding author
Address correspondence to: Jean C. Shih
Department of Pharmacology and Pharmaceutical Sciences, School of Pharmacy, University of Southern California, Los Angeles, CA 90089, USA. Phone: 323-442-1441; E-mail: jcshih@usc.edu
Address correspondence to: Pei-Ching Chang
Institute of Microbiology and Immunology, National Yang-Ming University, Taipei 11221, Taiwan. Phone: +886-2-2826-7111; E-mail: pcchang@ym.edu.tw

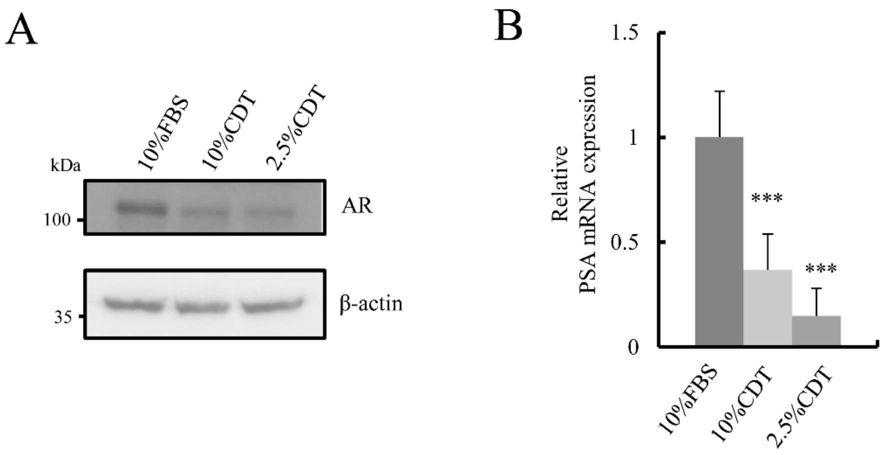

Supplementary Figure S1. Expression of the androgen receptor (AR) and prostate-specific antigen in androgen (PSA) deprivation-induced NE differentiated LNCaP cells.

(A) Protein expression of AR in LNCaP cells cultured with 10% FBS, 10% or 2.5% CDT for 96 hours was detected by Western blot.

(B) RNA level of PSA was measured in LNCaP cells treated as described in (A) using RT-qPCR. Data represent mean±S.D. (n=3); ****p*<0.001 by *Student's-t* test.

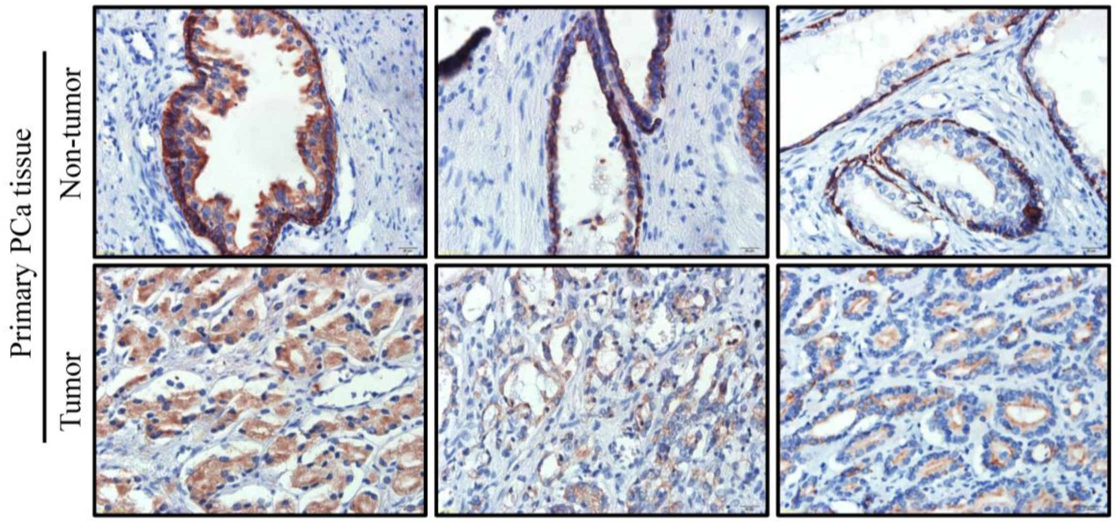

Supplementary Figure S2. Examples of IHC staining for basal cytokeratin 5 in six primary PCa specimens. Primary tumor (lower panel) and adjacent non-tumor tissue (upper panel) images are shown (magnification, x40).

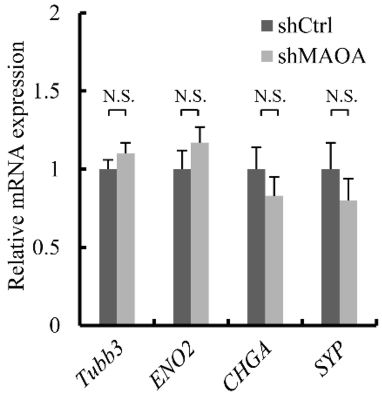

Supplementary Figure S3. Knockdown of MAOA does not affect the NED of LNCaP cells maintained in 10% FBS control medium.

The relative mRNA levels of NE marker genes in LNCaP-shCtrl and -shMAOA cells were assessed using RT-qPCR and normalized against 18S rRNA. Data represent mean±S.D. (n=3); N.S., not significant by *Student's* test.

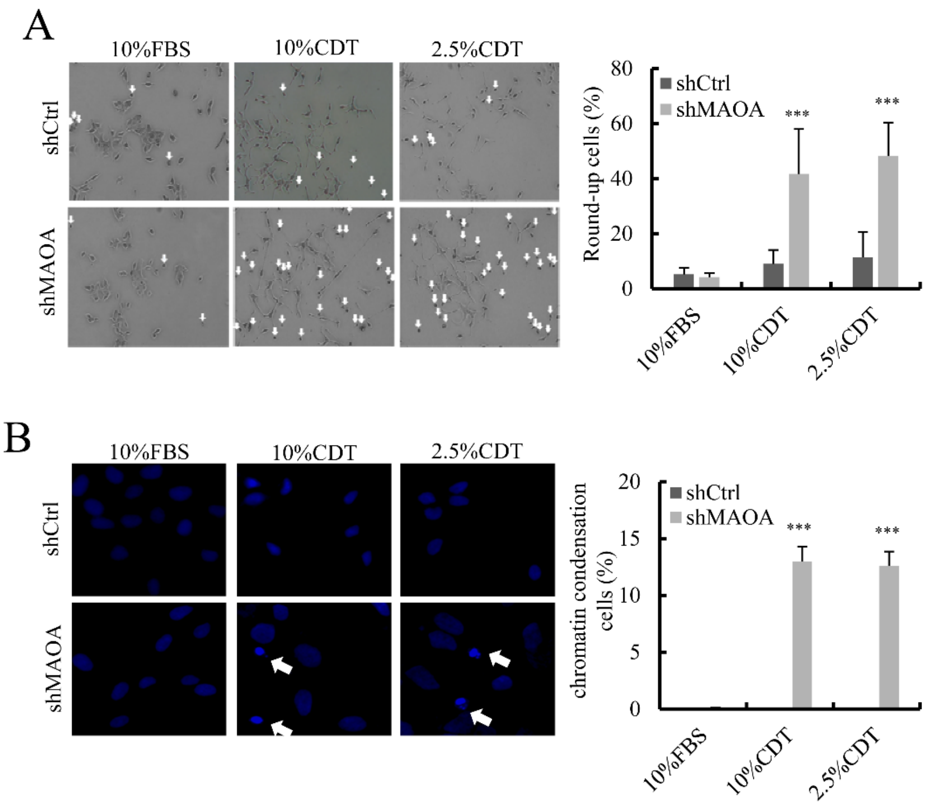

Supplementary Figure S4. Knockdown of MAOA results in an increase of NE differentiated cells with an apoptotic phenotype.

(A) MAOA knockdown increased the round-up cell number in LNCaP cells treated with 10% or 2.5% CDT for 96 hours. Representative photos (50x magnification) are shown in the left panel and the number of round-up cells was quantified using the average from 200 to 300 cells in random fields from three independent experiments. Data represent mean±S.E.M. ***$p<0.001$ by *Student's-t* test.

(B) Knockdown of MAOA increased the number of cells containing condensed chromatin. Cells were treated as described in (A). Representative photos of DAPI staining are shown in the left panel and the percentage of cells with chromatin condensation was quantified by counting a minimum of 100 cells in random fields. White arrows indicate cells with condensed chromatin. Data represent mean±S.E.M. ***$p<0.001$ by *Student's-t* test.

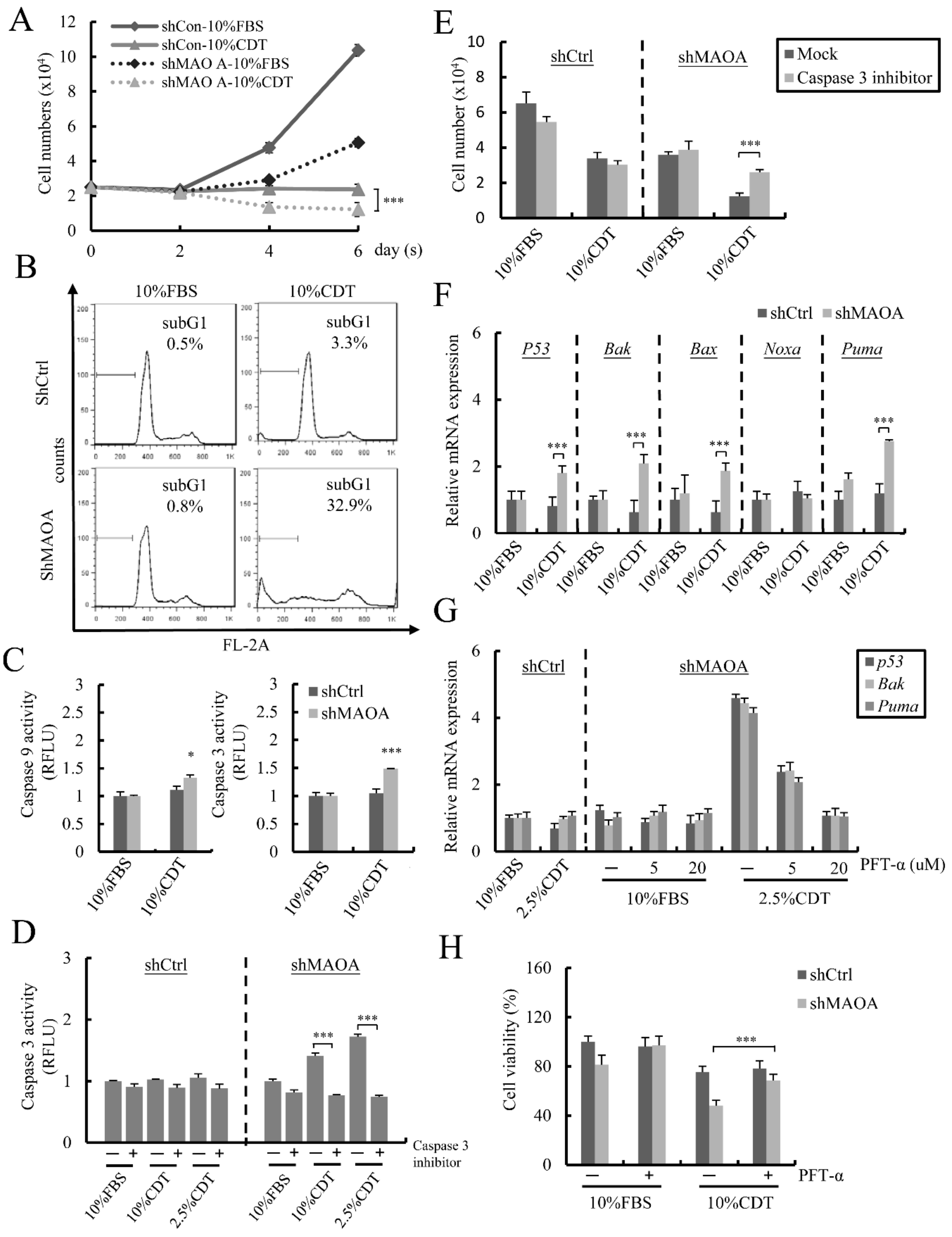

Supplementary Figure S5. Knockdown of MAOA induces p53-dependent cell apoptosis of 10% CDT-treated NE differentiated LNCaP cells.

(**A**) LNCaP-shCtrl and -shMAOA cells treated with 10% CDT were evaluated at indicated time points using trypan blue dye exclusion assay. Data represent mean±S.D. (n=3); ****p*<0.001 by

Student's-t test.

(**B**) The subG1 population of cells treated as described in (A) for 96 hours. Cells were stained with propidium iodide and analyzed by flow cytometry. (n=2)

(**C**) Cytosolic caspase 3 and 9 activity were measured using fluorogenic caspase substrates in cells treated as described in (A) for 96 hours. Data represent mean$\pm$S.D. (n=3); *$p<0.05$, ***$p<0.001$ by *Student's-t* test.

(**D**) Successful inhibition of caspase 3 activity in control and MAOA knockdown LNCaP cells cultured in 10% or 2.5% CDT for 96 hours with Q-DEVD-OPh was determined. Data represent mean$\pm$S.D. (n=3); ***$p<0.001$ by *Student's-t* test.

(**E**) Effect of caspase 3 inhibition by 5 µM Q-DEVD-OPh on the growth of cells treated and measured as described in (C). Data represent mean$\pm$S.D. (n=3); ***$p<0.001$ by *Student's-t* test.

(**F**) Expression of intrinsic apoptosis pathway genes was examined in cells treated as described in (A) using RT-qPCR and normalized with β-actin. Data represent mean$\pm$S.D. (n=3); ***$p<0.001$ by *Student's-t* test.

(**G**) Successful inhibition of p53 and p53-mediated transactivation of its target genes in MAOA knockdown LNCaP cells cultured in 10% or 2.5% CDT for 96 hours by PFT-α was determined using RT-qPCR.

(**H**) Inhibition of p53 by 20 µM PFT-α increased viability of LNCaP-shMAOA cells treated as in (G). Data represent mean$\pm$S.D. (n=3); ***$p<0.001$ by *Student's-t* test.

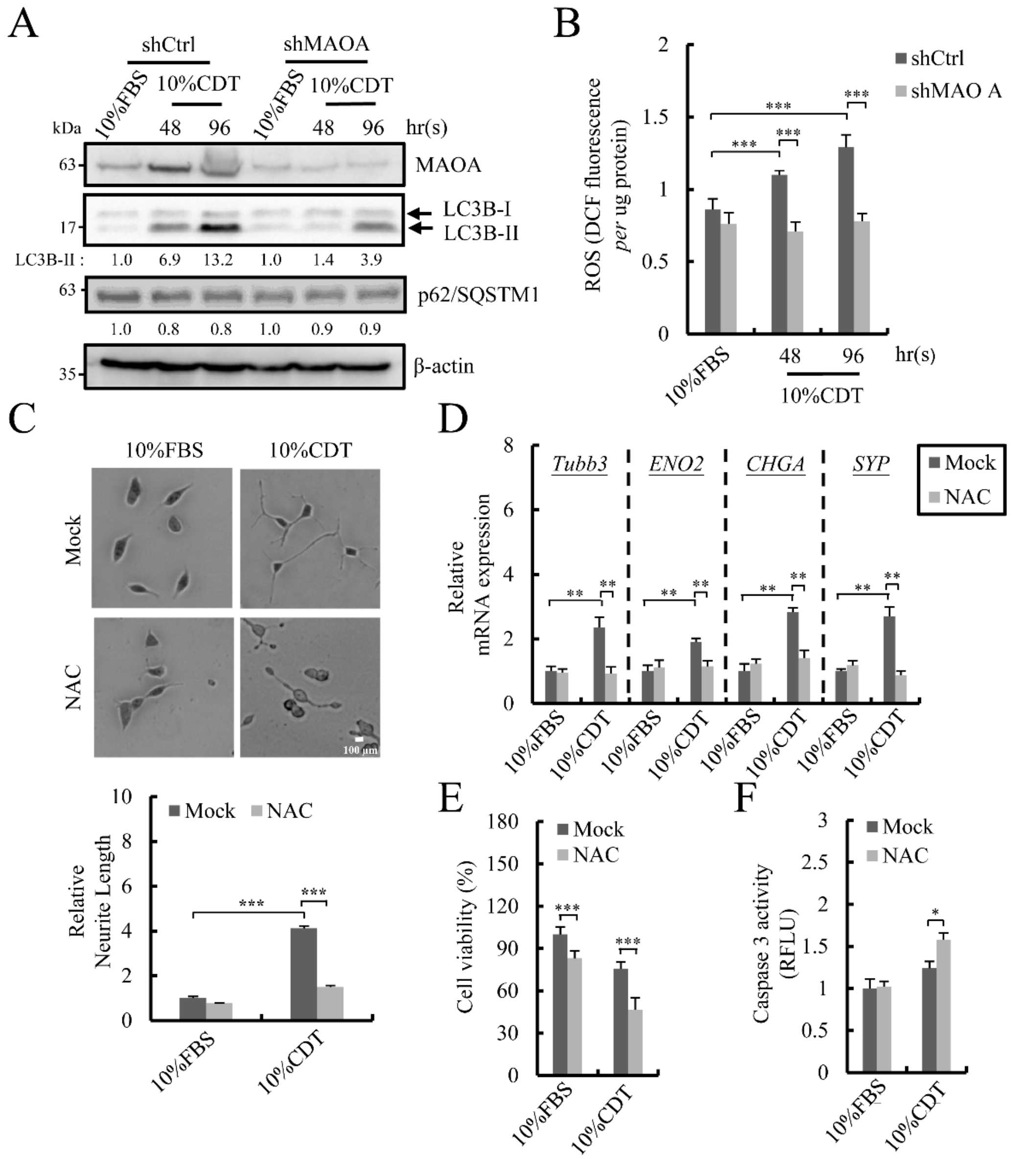

Supplementary Figure S6. ROS produced by MAOA plays an essential role for 10% CDT-induced autophagy activation, NED and anti-apoptosis.

(**A**) Immunoblotting of MAOA, LC3B-II and p62/SQSTM1 in LNCaP-shCtrl and -shMAOA cells treated with 10% CDT for 48 and 96 hours. β-actin was used as loading control. Uncropped images are presented in Supplementary Figure S12A (left).

(**B**) ROS production measured by OxiSelect *In Vitro* ROS/RNS Assay Kit in cells treated as described in (A). Data represent mean±S.D. (n=3); ****p*<0.001 by *Student's-t* test.

(**C and D)** Inhibition of ROS reduces neurite extension (C) and expression of NE markers (D) in LNCaP cells treated with 10% CDT in the presence or absence of 5 mM NAC for 96 hours. Data represent mean±S.D. (n=3); ***p*<0.01, ****p*<0.001 by *Student's-t* test.

(**E and F**) Inhibition of ROS decreased cell viability (E) and increased caspase 3 activity (F) of LNCaP cells treated as described in (C). Data represent mean±S.D. (n=3); **p*<0.05, ****p*<0.001 by *Student's-t* test.

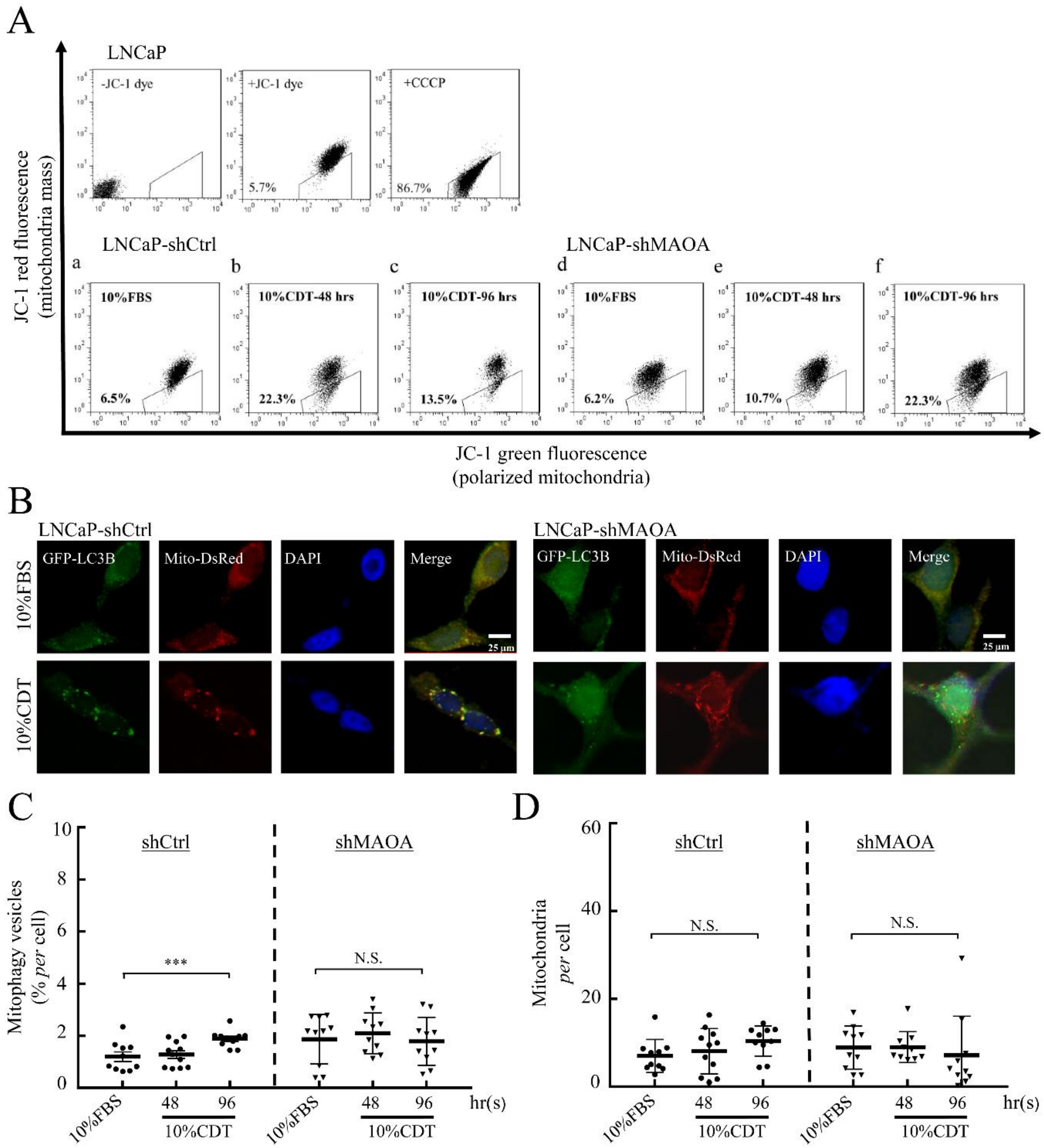

Supplementary Figure S7. Effect of MAOA on the loss of mitochondrial membrane potential and mitophagy activation of 10% CDT-induced NE differentiated LNCaP cells.

(**A**) Flow cytometry analysis of LNCaP cells with no staining (upper left), JC-1 staining (upper middle, negative control), and JC-1 staining after treatment with carbonyl cyanide 3-chlorophenylhydrazone (CCCP) for 5 minutes (upper right, positive control). LNCaP-shCtrl and -shMAOA cells were treated with 10% CDT for 48 and 96 hours and stained with JC-1. JC-1 fluorescence was measured in FL-1 (green, representing the total mitochondrial mass) and FL-2 (red, representing polarized mitochondria) channels. JC-1 fluorescence was detected in both FL-1 and FL-2 channels in the majority of LNCaP-shCtrl and -shMAOA cells cultured in 10% FBS, with a small percentage of cells showing reduced red fluorescence (a and d). 10% CDT treatment

increased the number of cells devoid of red fluorescence (b, c, e and f), indicating loss of mitochondrial membrane potential. (n=3)

(**B**) Representative fluorescence images of LNCaP-shCtrl and -shMAOA cells stably expressing GFP-LC3B, transiently transfected with mito-DsRed plasmid and treated by 10% CDT for 96 hours. Mitophagy was determined by assessing colocalization of GFP puncta, representing autophagosomes, and RFP puncta, representing mitochondria, using fluorescence microscopy. GFP and RFP double positive puncta with 0.8 to 1.3 μm in diameter were counted as mitophagy-positive cells.

(**C-D**) Quantification of the percentage of GFP (autophagosome) and RFP (mitochondrial) colocalized puncta in RFP puncta (C) and mitochondrial counts per cell (D) in cells treated as in (B). Data represent mean±S.E.M. Average value was quantified from 170 cells in random fields of three independent experiments. N.S., not significant, ***p<0.001 by *Student's-t* test.

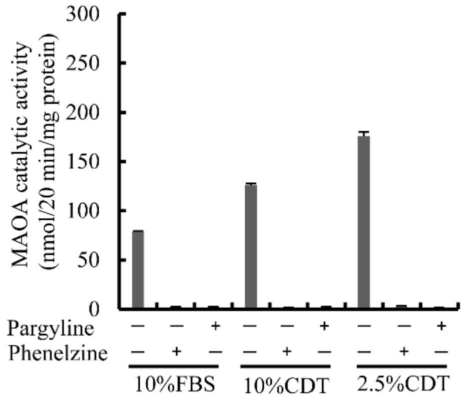

Supplementary Figure S8. Successful inhibition of MAOA activity in LNCaP cells by MAOA inhibitors pargyline and phenelzine.

MAOA activity in LNCaP cells treated with 10% or 2.5% CDT in the presence or absence of pargyline (1 µM) and phenelzine (1 µM) for 96 hours was determined using MAOA catalytic activity assay with [^{14}C]5-HT substrate.

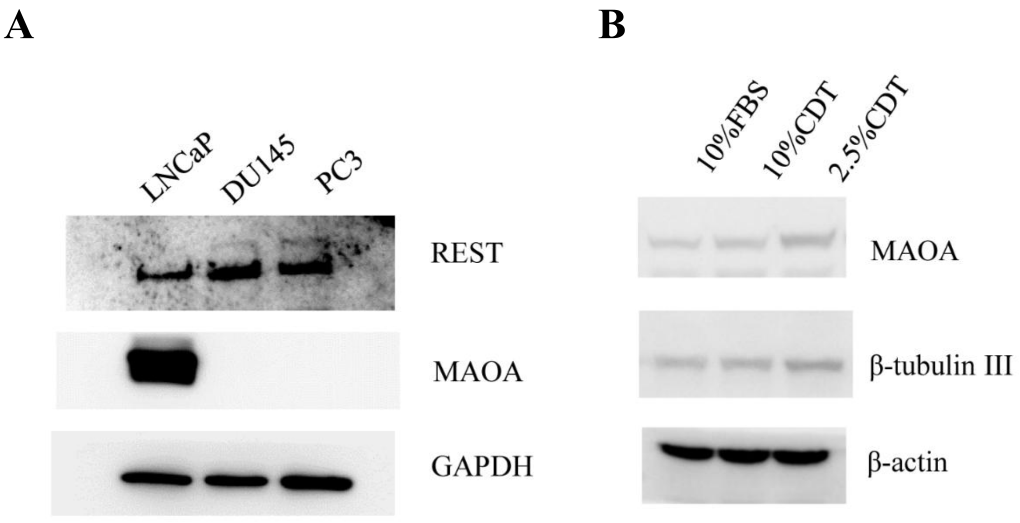

Supplementary Figure S9. Uncropped versions of the blots in (A) Figure 1A and (B) Figure 1D.

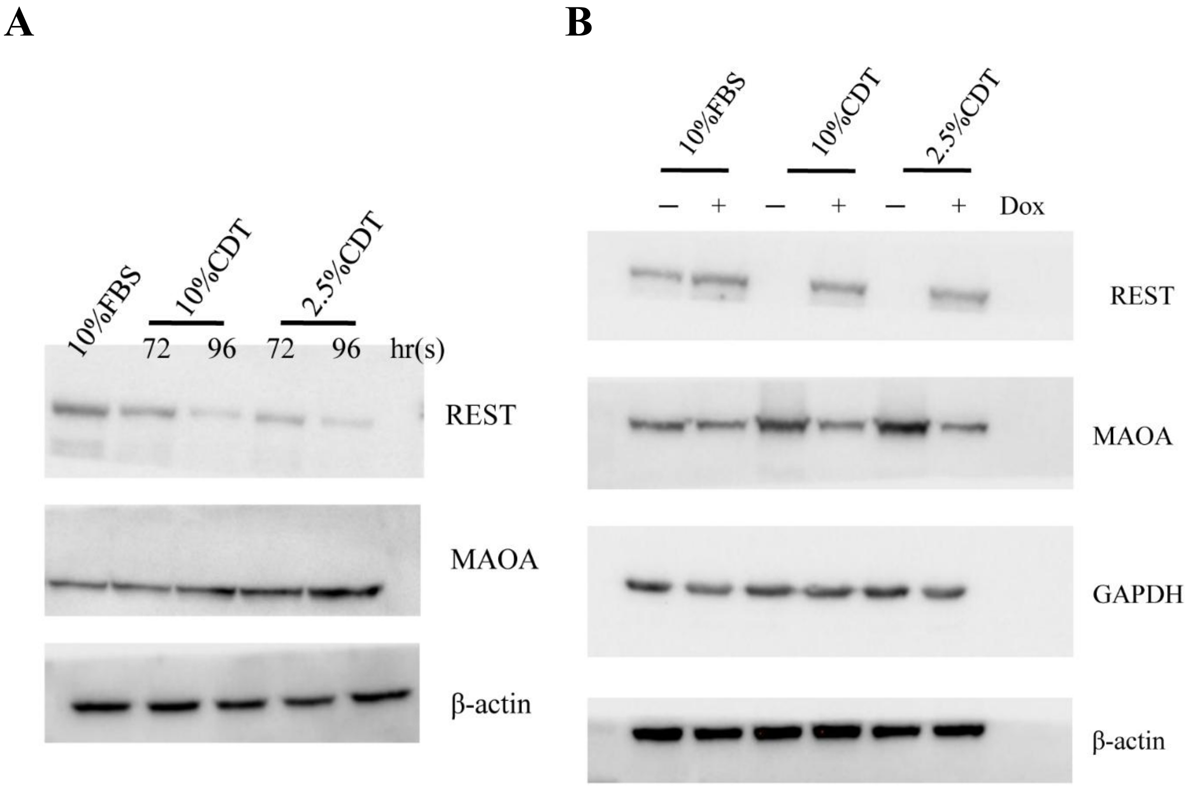

Supplementary Figure S10. Uncropped versions of the blots in (A) Figure 2D and (B) Figure 2F.

A

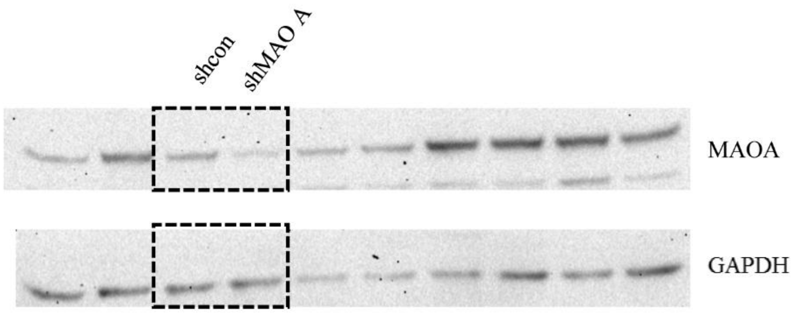

B

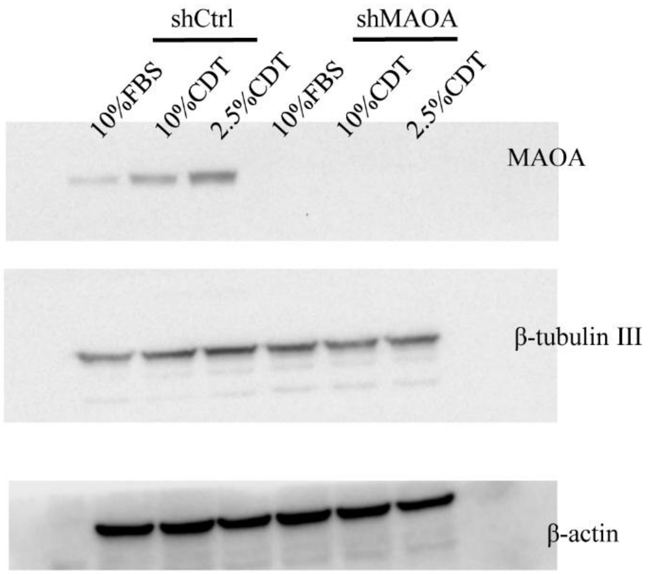

Supplementary Figure S11. Uncropped versions of the blots in (A) Figure 3A and (B) Figure 3E.

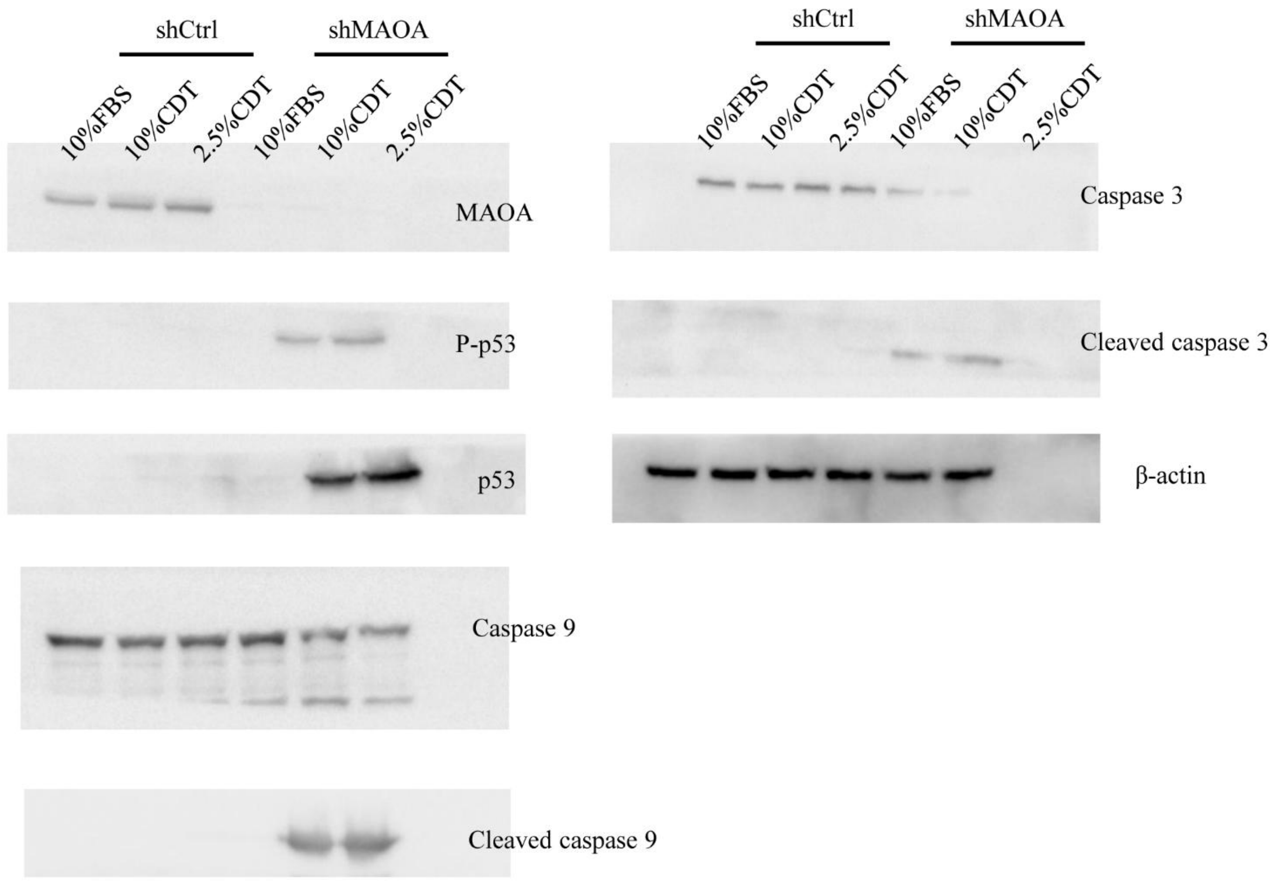

Supplementary Figure S12. Uncropped versions of the blots in Figure 4D.

A

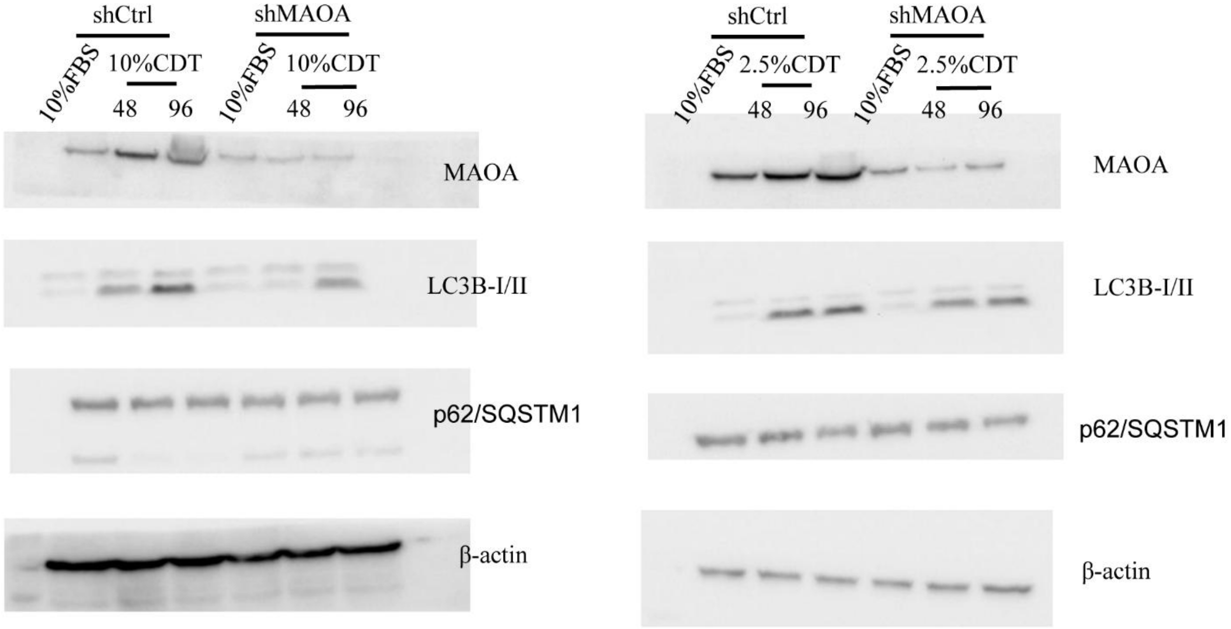

B

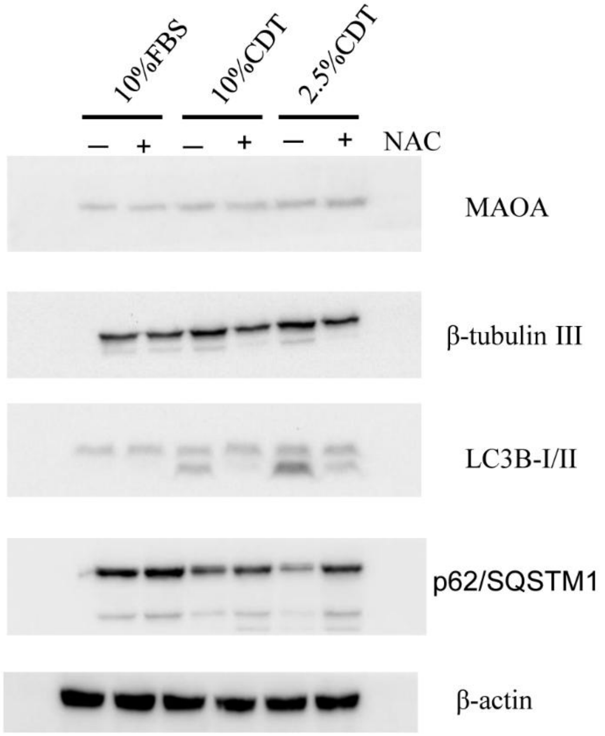

Supplementary Figure S13. Uncropped versions of the blots in (A) Supplementary Figure 5A (left) and Figure 5A (right) and (B) Figure 5C.

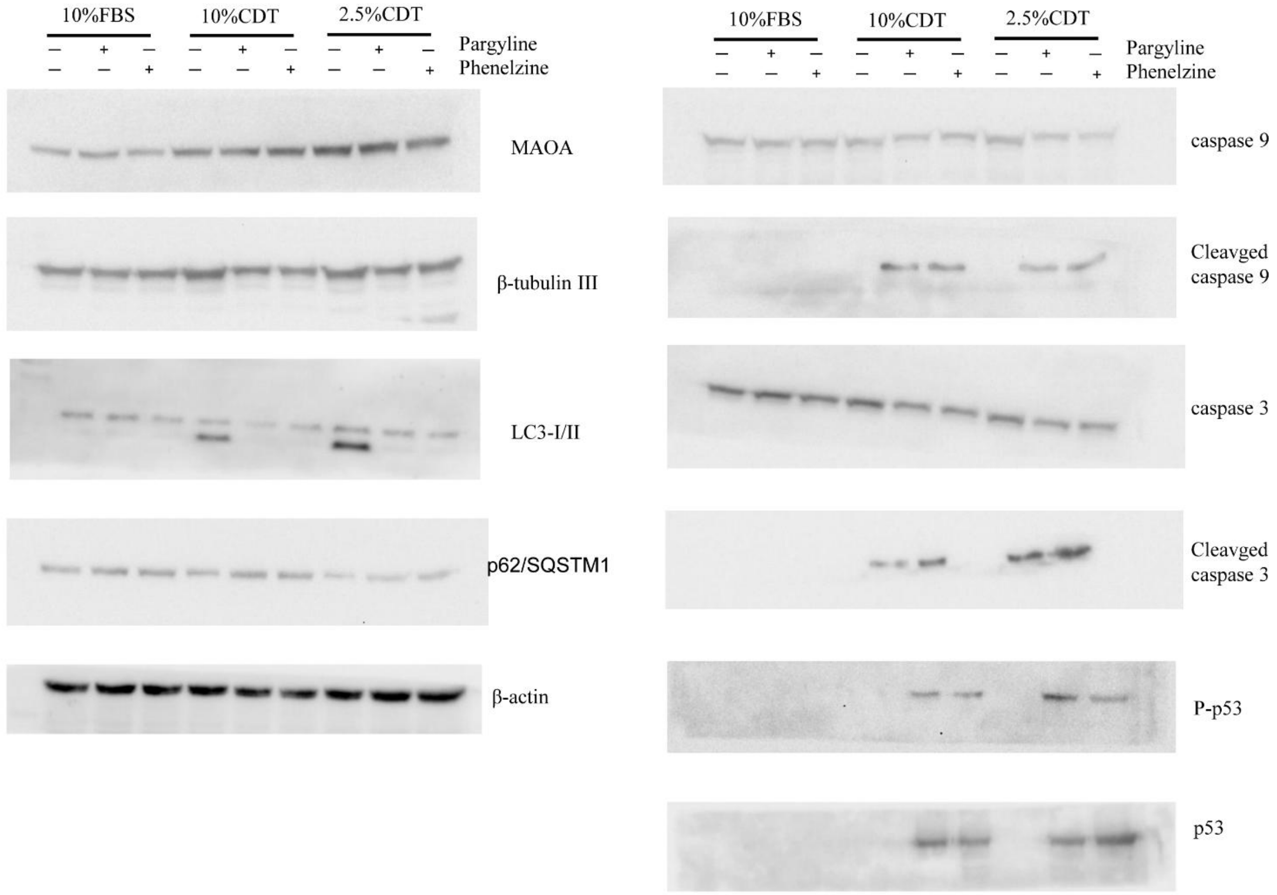

Supplementary Figure S14. Uncropped versions of the blots in Figure 7C.

Supplementary Table 1. Primer sequences used for real-time RT-qPCR.

Target gene	Forward primer	Reverse primer
TUBB3	CTCTTCTCACAAGTACGTG	CCCACTCTGACCAAAGATG
CHGA	ATACCGAGGTGATGAAATGC	AGGATCCGTTCATCTCCTC
ENO2	GCCAAAGGTCTTTTCCGG	CCTTCAGGACACCTTTGC
SYP	CTACCAGCCTGACTATGG	GGGCTTCACTGACCAGAC
MAOA	CAGTGGCAGGGTTTCCAG	CACGGTCAGGCTGTTCTCG
RNA18S5	CGCCGCTAGAGGTGAAATTC	CGAACCTCCGACTTTCGTTC
ACTB	ACGTTGCTATCCAGGCTGTGCTAT	TTAATGTCACGCACGATTTCCCGC
TP53	CAGCCAAGTCTGTGACTTGCA	GTGTGGAATCAACCCACAGCT
BAK	TGGAGCTGCAGAGGATGATTG	GAAGTTGCCGTCAGAAAACAT
BAX	GAGAGGTCTTTTTCCGAGTGG	CCTTGAGCACCAGTTTGCTG
Puma	GGACGACCTCAACGCACAGT	AATTGGGCTCCATCTCGGGG
Noxa	GAGATGCCTGGGAAGAAGG	CCTGAGTTGAGTAGCACACTCG
